# Chromate-Free Corrosion Protection Strategies for Magnesium Alloys—A Review: PART I—Pre-Treatment and Conversion Coating

**DOI:** 10.3390/ma15238676

**Published:** 2022-12-05

**Authors:** Bahram Vaghefinazari, Ewa Wierzbicka, Peter Visser, Ralf Posner, Raúl Arrabal, Endzhe Matykina, Marta Mohedano, Carsten Blawert, Mikhail Zheludkevich, Sviatlana Lamaka

**Affiliations:** 1 Institute of Surface Science, Helmholtz-Zentrum Hereon, 21502 Geesthacht, Germany; 2 Departamento de Ingeniería Química y de Materiales, Facultad de Ciencias Químicas, Universidad Complutense de Madrid, 28040 Madrid, Spain; 3 Department of Functional Materials and Hydrogen Technology, Faculty of Advanced Technologies and Chemistry, Military University of Technology, 2 Kaliskiego Street, 00-908 Warsaw, Poland; 4 AkzoNobel, 2171 AJ Sassenheim, The Netherlands; 5 Henkel AG & Co., KGaA, 40589 Düsseldorf, Germany

**Keywords:** pre-treatment, conversion coating, corrosion, magnesium, hexavalent chromium

## Abstract

Corrosion protection systems based on hexavalent chromium are traditionally perceived to be a panacea for many engineering metals including magnesium alloys. However, bans and strict application regulations attributed to environmental concerns and the carcinogenic nature of hexavalent chromium have driven a considerable amount of effort into developing safer and more environmentally friendly alternative techniques that provide the desired corrosion protection performance for magnesium and its alloys. Part I of this review series considers the various pre-treatment methods as the earliest step involved in the preparation of Mg surfaces for the purpose of further anti-corrosion treatments. The decisive effect of pre-treatment on the corrosion properties of both bare and coated magnesium is discussed. The second section of this review covers the fundamentals and performance of conventional and state-of-the-art conversion coating formulations including phosphate-based, rare-earth-based, vanadate, fluoride-based, and LDH. In addition, the advantages and challenges of each conversion coating formulation are discussed to accommodate the perspectives on their application and future development. Several auspicious corrosion protection performances have been reported as the outcome of extensive ongoing research dedicated to the development of conversion coatings, which can potentially replace hazardous chromium(VI)-based technologies in industries.

## 1. Foreword

Corrosion dramatically impacts the economics and ecology of a wide range of global infrastructure aspects. Industries across the globe are therefore obligated to spend astronomical amounts of money annually to prevent and treat corrosion. Thus, researchers have been placing a great deal of attention on corrosion and corrosion prevention over the last several decades.

Chromate-based surface treatments have been one of the most robust and effective technologies to prevent the corrosion of different metals including magnesium and its alloys. Their unique properties include enhancing the adhesion of subsequently applied organic coatings, providing an excellent corrosion barrier, and featuring ”self-healing” characteristics [1]. However, chromate is notorious for its toxicity and carcinogenicity [2,3]. Therefore, the elimination of chromate from surface treatment processes is highly sought after in order to reduce work-related health risks and avoid environmental harm. This has led to the ban or restricted use of chromate in industrial applications [4].

Consequently, the focus of extensive efforts in recent years has been to develop environmentally friendly corrosion protection alternatives that are comparable or superior to chromate-based corrosion protection technologies including conversion coatings, plasma electrolytic oxidation (PEO) coatings, surface pre-treatments, and corrosion inhibitors.

This review is the first part of a trilogy on Cr(VI)-free corrosion protection strategies for magnesium alloys. This part focuses on the alternative chromate-free pre-treatment and conversion coating systems for magnesium and its alloys. These two treatments must be addressed together, as the pre-treatment step that aims to modify the magnesium surface properties plays a critical role in the performance of the subsequent conversion layer against corrosion. **PART II** of the review [5] focuses on Plasma Electrolytic Oxidation (PEO) coating as one of the highly developed methods to protect magnesium surface in the recent years. **PART III** [6] reviews corrosion inhibitors for magnesium and approaches to incorporate them into coating systems. An overview of the review trilogy is illustrated in the graphical abstract of all three review parts.

## 2. Surface Cleaning and Pre-Treatment

### 2.1. Introduction

The most common strategy to prevent the corrosion of metallic materials in industry is the application of layers of coatings that protect the metal surface from the external corrosive species. The general schematic of this system of layers is shown in the pictogram above. The corrosion protection is provided via either a barrier effect or an active corrosion suppression or their combination. Prior to the application of these protective layers, the metal surface must be modified through a single or a series of pre-treatment steps. Although a pre-treatment step can dramatically affect the corrosion protection properties of the substrate protected by a coating system, much less attention has been accorded to it compared to the coating system itself. 

The pre-treatment steps have two main objectives that lead to the enhancement of the corrosion properties of the coated metal system:

1. Improvement in the corrosion resistance of the metallic substrate.
2. Preparation of an adequate surface for the subsequent layers in the coating system.

Both the above-mentioned objectives are fulfilled by modifications of the surface characteristics including the contamination level, microstructure, roughness, composition, and morphology. Each of the mentioned surface characteristics impacts the properties of the protective coating system in various ways, and therefore should be optimized according to the desired coating criteria. Moreover, the fulfillment of each of the objectives does not necessarily lead to a decrease in the overall corrosion rate of the coated substrate. As a quick example, it is well-known that the higher roughness of the substrate enhances the mechanical interlocking, leading to higher adhesion between a coating and a substrate [7,8]. However, a rougher surface may lead to a higher corrosion rate of the bare substrate [9,10,11].

Any contamination on the surface leads to inefficiency of the coating materials or their precursors to reach the substrate, which leads to weaker adhesion between the coating and the substrate. Furthermore, conversion coatings typically require access to metallic magnesium rather than the native layer of MgO/Mg(OH)_2_. Therefore, any level of contamination on the surface leads to coating non-uniformity and lower quality. Common contaminations can be enumerated as oil, grease, lubricant, dust, or other metallic particles that might be left on the surface during the production process. Contamination removal can be conducted with various pre-treatment methods, which can be sorted into two main categories: mechanical and chemical pre-treatment.

### 2.2. Mechanical Pre-Treatment

Based on the production history, surface condition, and geometry of the magnesium substrate, a proper mechanical pre-treatment method including sandblasting, grinding, and machining can be chosen to remove the contaminations from the surface.

Mechanical cleaning is designed to rapidly remove the outer layers of the material, regardless of the microstructure and chemical composition of the surface. The material removal can be carried out in the range of several millimeters to a few microns depending on the tools employed. Although mechanical cleaning appears to be a rough approach to removing contaminations or discontinuities from the surface, it must be conducted with high precision and sufficient care. All mechanical processes can easily impose plastic deformation on the magnesium surface, even several millimeters in the thickness, leading to the formation of twinning and dislocations that may be undesirable for the corrosion resistance properties of magnesium [12]. Moreover, mechanical processes can introduce further contamination by transferring from the corresponding mechanical tool to the magnesium surface.

**Machining**, apart from its primary use to guarantee the dimension of the components, is mainly used to remove physical discontinuities such as porosities due to the casting processes and rough finishing due to the wrought deformation processes. Therefore, enhancement of the corrosion properties of the part is not the main goal of the machining process, and it may even have adverse effects on the surface condition by introducing detrimental contamination transferred from the machining tools (typically made of steels) onto the magnesium surface. 

**Blasting** is a common mechanical pre-treatment technique in the industry that is employed to remove paint, dirt, and contamination from the metal surface. It is a popular method in the industry due to its fast and convenient process and universal applicability to various metallic substrates. However, adverse effects of sandblasting on corrosion resistance have frequently been reported for Mg and its alloys [13,14,15,16]. The reason for these adverse effects is mainly attributed to the contamination introduced to the magnesium surface during sandblasting. Sand particles, if they have been previously used to blast steel parts, can pick up Fe-rich particles from steel and deposit them on the Mg surface. An increase in the level of Fe-rich impurities on AZ31 after sandblasting has been confirmed via energy dispersive X-ray spectroscopy (EDS) from the treated Mg surface [14]. Furthermore, since magnesium is a relatively soft metal, sand particles can be stuck onto the surface and act as the contamination themselves [14,17]. Micro-stress imposed on the magnesium surface is another reason attributed to the high corrosion rate of the magnesium alloy after sandblasting [14,15]. It has also been reported that sandblasting can induce the recrystallization of the β-phase near the surface of the AZ91D alloy. The recrystallized β-phase acts as cathodic sites with respect to the alpha Mg matrix, leading to micro-galvanic corrosion [13]. Despite the commonly reported adverse effect of sandblasting on the corrosion resistance of bare Mg, a higher rate of deposition in the electroless Ni plating on the AZ31 Mg alloy after pre-treatment with alumina blasting compared to the ground surface with SiC paper has been observed [18,19], which is attributed to the more favorable sites for the nucleation of the Ni–P coating. Nevertheless, a proper corrosion resistance evaluation of such coatings is not available. 

**Grinding** is another mechanical pre-treatment method that is used to remove the outer layers of the surface. With this method, the roughness of the surface can be controlled over a wide range of values. There are many investigations reporting the positive effects of grinding pre-treatment on the corrosion protection properties of magnesium alloys compared to the as-received samples [9,11,13,14], which is mainly attributed to the removal of the contaminations, especially detrimental Fe-rich contaminations from the surface. Moreover, the roughness of the surface, which can be controlled in the grinding method, plays an important role in the corrosion properties of bare and coated magnesium alloys. 

**Figure 1:**
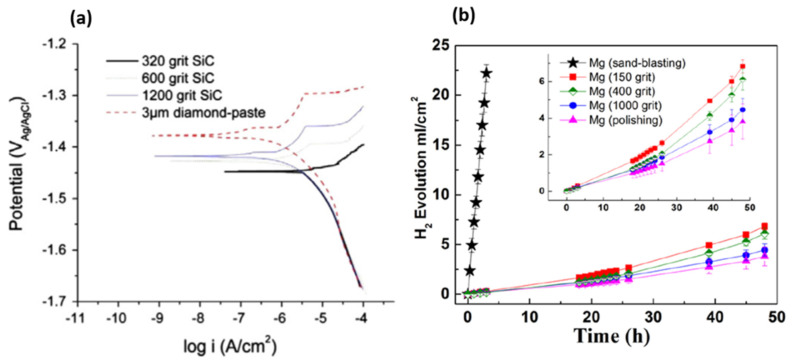
(**a**) Potentiodynamic polarization curves of the AZ91 alloy, with different surface roughness, tested in 0.5 wt.% NaCl [9]. (**b**) Hydrogen evolution of the pretreated AZ91 alloy in 3.5 wt. % NaCl solution [13]. Reprinted from [9] and [13] with permission from Elsevier.

Walter et al. [9] produced different roughness on an AZ91D magnesium alloy using SiC grinding papers with different grit sizes (i.e. 320, 600, and 1fI00) and by polishing with 3 µm diamond paste. The potentiodynamic test in 0.5 wt.% NaCl solution (Figure 1) illustrated the direct relationship between the surface roughness and the corrosion current. The anodic activity of the magnesium surface was considerably reduced for a less rough surface, while the cathodic activity remained at a similar value for all surface roughness. Moreover, an increase in the surface roughness leads to the loss of the passivation behavior in the anodic polarization region. In another work by Zhang et al. [13], a higher H_2_ evolution rate on the AZ91D alloy with higher roughness confirmed the adverse effect of surface roughness on the corrosion resistance of the Mg surface (see Figure 1). 

**Figure 2:**
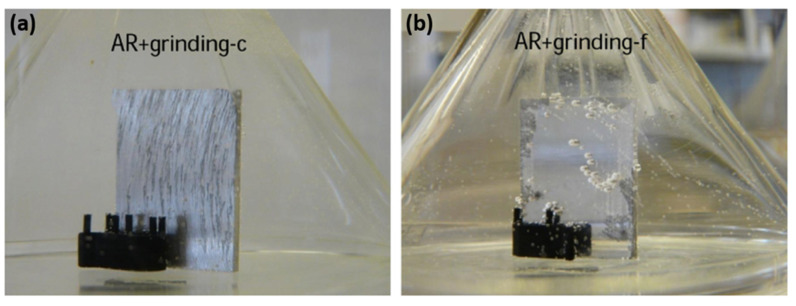
The in situ observation of the corrosion morphologies of the AZ31 magnesium sample ground with (**a**) SiC paper (marked as AR+grinding-c); (**b**) polished up to 1 µm diamond paste (marked as AR+grinding-f) in 5 wt.% NaCl [14]. “AR” stands for “as-received”. Reprinted from [14] with permission from Elsevier.

However, it must be taken into account that the surface condition prepared by different emery paper grit numbers is not merely reflected by its roughness, which is only a geometrical parameter. In fact, the residual stress stored in the surface layer varies when different emery papers are used for the grinding of the surface. Generally, the higher roughness produced by emery paper leads to a higher level of stress stored in the surface. Assuming a ridge-valley morphology on the surface produced by the grinding process, the microstructural deformation at the bottom of the valleys is higher than the tip of the ridges [13], which leads to higher electrochemical activity at the valleys [20,21,22]. The evidence for this is shown by the black traces of corrosion on the ground magnesium surface, which were similar to the grinding patterns, as seen in Figure 2. However, when the surface was polished up to 1 µm diamond paste, there was no evidence of the grinding marks. Further details about the effect of surface roughness on the corrosion properties of magnesium/coating systems are given in the chemical pre-treatment section.

Note that all mechanical cleaning methods that remove layers from Mg surfaces and produce fine magnesium particles need to be carefully considered to avoid ignition or explosion. Magnesium powder is highly flammable, and the ignition temperature decreases with the decrease in the magnesium particle size. For instance, magnesium particles with a size of 6 µm ignite at temperatures as low as 377 °C [23,24]. Therefore, the use of a fluid that controls the temperature near the machining zone and removes the fine particles is always recommended during mechanical pre-treatment methods [25]. In addition, it has been reported that a dry-abrading mechanical cleaning method exhibits lower corrosion resistance than a wet-abrading mechanical cleaning method when an E-coating is applied to the AZ31 alloy [26].

**Other mechanical treatments**

There are other mechanical surface treatments that are often utilized to modify the microstructure of the Mg surface rather than aiming for the impurity removal or chemical composition modification of the Mg surface. These techniques including shot peening, ultrasound shot peening (USSP), laser shock peening (LSP), surface mechanical attrition treatment (SMAT), and ball burnishing, etc. are primarily designed to improve the tribological properties of the metallic part by inducing near-surface compressive residual stress [27,28,29,30,31]. However, the investigation of the corrosion resistance influenced by these strategies has also shown that these techniques can be exploited for the simultaneous improvement in the fatigue and degradation life of magnesium structures [32,33,34,35,36,37,38,39].

The mentioned surface mechanical pre-treatments involve a severe deformation on the surface of Mg, which leads to a higher compressive residual stress, a smaller grain size, modification in the roughness, nucleation/dissolution of second phases, and a crystallographic texture [35,36,37,38,40,41,42]. All of these effects can change the surface electrochemical activity and thus the corrosion resistance.

For instance, one order of magnitude reduction in the current density of AZ31 magnesium alloys in 3.5 wt.% NaCl after LSP pre-treatment has been reported [39]. The enhancement in the corrosion resistance of the treated alloy was attributed to the increased grain boundaries and compressive residual stress. In another work by Zhu et al. [35], the lower corrosion rate of a burnished pure Mg compared to the untreated Mg was attributed to the generated strong basal texture. The increase in grain boundary density after burnishing was also believed to help dissolve the impurities and thus reduce the number of micro-galvanic cells. However, surface mechanical pre-treatments like LSP can simultaneously modify several microstructural characteristics of the Mg surface, each of which may have an adverse or positive effect on the corrosion resistance. This has been, for instance, reflected in the contradictory reports on the effect of individual microstructural parameters such as grain orientation and grain size [43].

The microstructure and morphology of the Mg surface tailored by the treatment procedure also determine the characteristics of the subsequent conversion coating. Zhang et al. [13] showed that the peak-to-valley surface morphology produced by grinding could lead to a locally different chemical composition of a phosphate conversion layer. Thus, a more uniform and less porous phosphate conversion layer with superior corrosion resistance can form on a smoother Mg surface.

In a recent study by Uddin et al. [34], a mechanical pre-treatment on AZ31 by burnishing methods was shown to result in a denser subsequent hydroxyapatite (HA) deposition compared to that formed on the untreated AZ31. The modification in the HA morphology was attributed to the higher nucleation rate on the modified surface due to its higher electrochemical activity. Analogously, the modified surface of AZ31 after LSP pretreatment led to a thicker and denser subsequent phosphate conversion coating [39].

On the other hand, the adverse effect of mechanical pre-treatments such as shot peening [32], USSP [30,31], and SMAT [44,45,46,47] on the corrosion resistance of different magnesium alloys has frequently been observed. Interestingly, the inferior corrosion resistance observed after the majority of the treatment types for several different alloys (pure Mg, AZ31, AZ91D, and Mg1Ca) is due to the significant increase in the cathodic activity of alloys evident from the dynamic polarization curves, albeit with only a slight variation in the corresponding anodic activity. High residual stress, high level of crystallographic defects such as dislocations and grain boundaries are the main reasons attributed to the adverse effect of shot peening and SMAT on Mg corrosion resistance. However, the introduction of Fe contamination during these processes, which involve the high-energy impact of stainless steel balls onto the Mg surface, should not be overlooked [30,31,48]. Additionally, the Fe contamination can be indirectly transferred from the SMAT chamber, even with the impacting balls made of alumina or zirconia [48].

**Figure 3:**
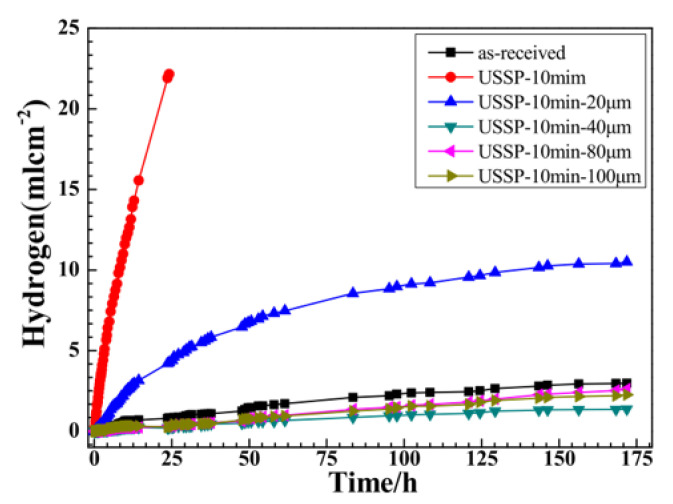
H_2_ evolution of AZ31 in NaCl 3.5 wt.% before and after a USSP process. The length mentioned after each case indicates the thickness removed by grinding using 2000 grit size SiC paper. Reprinted from [31] with permission from Elsevier.

Zhang et al. [31] showed that grinding of the SMATed AZ31 surface for more than 20 µm could significantly reduce the corrosion rate (Figure 3). Fe contamination up to a depth of 10 µm has also been reported on the surface of a pure Mg SMATed with steel balls [48].

The practicality of the mechanical methods is directly impacted by the geometry of the surface. If the geometry is complex, the uniform outreach to the entire surface might also be of high complexity. Although stand-alone mechanical cleaning has been reported to provide an improved surface condition, subsequent chemical pre-treatment is mostly inevitable [49,50].

### 2.3. Chemical Pre-Treatment

In contrast to mechanical pre-treatment, chemical pre-treatment can be easier to apply to complex geometries and targets specific contaminations or phases on the surface. Therefore, it can be conducted in different steps, each assigned to remove specific phases or contaminations. Moreover, chemical pre-treatment is not necessarily carried out to clean the magnesium surface from contaminations. It can be carried out exclusively with the aim of providing a surface condition that is desired for the formation of the subsequent conversion coating. Chemicals used for the pre-treatment step are commonly categorized into alkaline and acidic solutions. Organic solvents can also be used before the pre-treatment step in order to remove grease and oil left from the production procedure or mechanical pre-treatment. Acetone and ethanol are the most common solvents used in scientific studies. However, other solvents such as paint thinners, chlorinated solvents, and typical alkane (paraffin) baths can also be used [12,51].

#### 2.3.1. Alkaline Degreasing

Alkaline degreasing is mainly used for the removal of grease, oil, and lubricant. Sodium hydroxide is the main ingredient of most of the alkaline degreasing solutions used both in academic research and in the industry. Degreasing is carried out by the immersion of the magnesium part in a solution with a concentration of NaOH ranging between 40 and 50 g/L and at an elevated temperature (70–90 °C) to accelerate the chemical process. As a partially passive Mg(OH)_2_ layer is formed on the magnesium surface during immersion in the alkaline solution (pH above 10.4), a negligible amount of magnesium phase is removed during alkaline degreasing. However, the second phases in Mg alloys might be susceptible to corrosion in highly alkaline conditions. For instance, a considerable amount of the aluminum-rich β phase in the AZ91 magnesium alloy can dissolve in a highly alkaline pre-treatment solution, which leads to a slight weight loss [52]. Surfactants are also common additives to alkaline degreasing solutions used in the industry to reduce the surface tension and ease the escape of the generated hydrogen bubbles from the surface [12]. Moreover, *cathodic cleaning* (negative polarization) can speed up the cleaning process by accelerating the detachment of the surface contaminants and causing local agitation on the surface. In this technique, hydrogen bubbles are produced by applying a potential between −4 to −8 V to the magnesium part [12]. However, the danger of hydrogen embrittlement must be taken into account in this case [53].

Alkaline pre-treatment does not only aim to clean the Mg surface from contaminations, but can also be carried out to increase the concentration of OH^−^ on the surface. The OH^−^ concentration on the surface has been shown to effectively facilitate the formation of some conversion coatings such as Ti/Zr/Hf-base, Ca–P conversion coating, and conversion coatings formed by phytic acid [54,55,56,57,58,59,60] (see Section 3 for more details on these types of conversion coatings). In such cases, the alkaline pre-treatment is directly preceded by the conversion coating step.

#### 2.3.2. Acid Pickling

A thin layer of oxide/hydroxide is readily formed on the surface of magnesium as soon as it is exposed to the atmosphere or an aqueous environment. Moreover, during the manufacturing process, the magnesium part may be subjected to different types of corrosive media that promote the formation of the oxide/hydroxide layer. The formed oxide/hydroxide is normally non-uniform and partially blocks the magnesium surface. One of the primary objectives of acid pickling is to remove the original oxide/hydroxide layer that hinders the formation of a uniform subsequent conversion layer.

**Figure 4:**
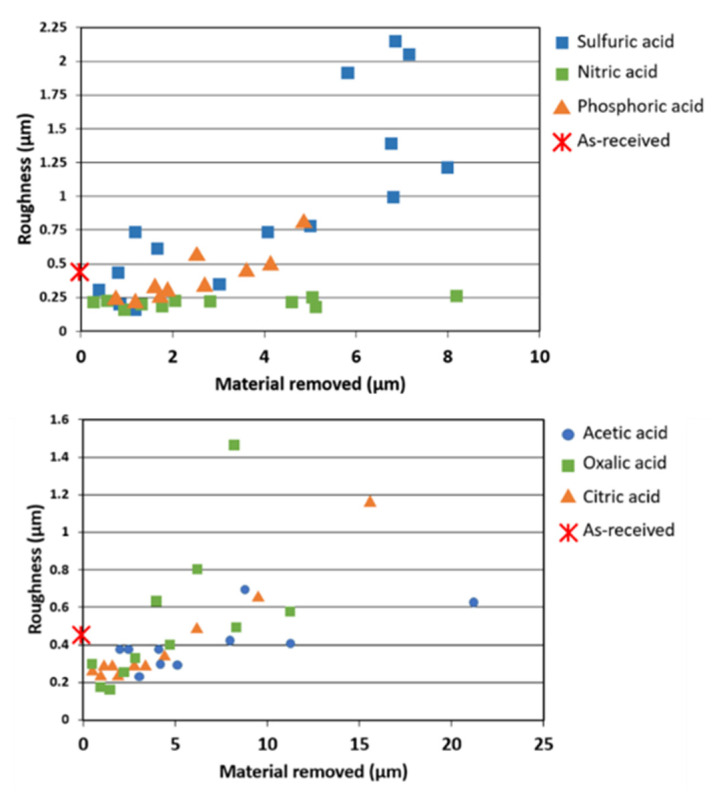
Variation in the surface roughness with material removed for different inorganic (**top**) and organic (**bottom**) pickling solutions [61,62]. Reprinted from [61,62] with permission from Elsevier.

When the magnesium part is immersed in an acid pickling solution, the oxide/hydroxide film on the surface dissolves. After fast removal of this film, the bare magnesium substrate is subjected to electrochemical oxidation, which is accompanied by the hydrogen evolution cathodic reaction. The dissolution rate of the magnesium is usually not uniform all over the surface due to the presence of heterogeneities in the microstructure such as second phases, impurities, and crystallographic heterogeneities including grain boundaries and orientations. Different dissolution rates of each of the mentioned heterogeneities lead to a change in the surface roughness. Moreover, several other parameters in an acid pickling step contribute to the final surface roughness including the time of etching, surface chemical/morphological properties of the magnesium substrate, and chemical composition of the etching solution. Nwaogu et al. [61,62] measured the roughness of the AZ31 alloy over the immersion time in organic acid pickling solutions of acetic acid, oxalic acid, and citric acid and in inorganic acid pickling solutions of phosphoric, sulfuric, and nitric acids. They observed that the roughness of the surface generally decreased initially compared to the as-received AZ31 alloy roughness (R_a_) of 0.44 ± 0.15 µm. Then, if the samples are kept immersed in the solution, a higher material removal results in a rapid increase in the roughness (see Figure 4). Among the tested inorganic acid pickling solutions, sulfuric acid resulted in the highest surface roughness, while nitric acid was able to remove the magnesium uniformly with a negligible change in roughness with immersion time.

Apart from the oxide/hydroxide layer, the aim is to remove the impurities and contaminations on the surface by acid pickling. The most common detrimental impurity element is iron, which can be found on the magnesium surface throughout the fabrication process such as machining or the sheet rolling process [63]. Even a small increase in the iron content on the surface or in the bulk can accelerate the corrosion of the magnesium alloy [64,65]. The acceleration in corrosion rate is significant when the Fe impurity level exceeds a value called the “corrosion tolerance limit”. The corrosion tolerance limit can vary from 5 ppm to more than 300 ppm, depending on the magnesium alloy microstructure, composition, and micro-constituents such as the Si amount [66,67]. 

Therefore, one of the most common ways to evaluate the effectiveness of the acid pickling step is to determine the amount of the material (or thickness) that is removed. This way, the removal of impurities on the surface can also be assured. Nwaogu et al. [61,62] compared the concentration of the impurity elements Fe and Ni on the surface when different organic and inorganic acid solutions were used to clean the AZ31 magnesium alloy. They observed that all inorganic acids such as sulfuric acid, nitric acid, and phosphoric acid were able to reduce the level of impurities on the surface. Moreover, they reported that organic acid solutions (i.e. acetic, oxalic, and citric acids) are also effective in the removal of Fe/Ni-containing impurities. Among the mentioned organic acids, acetic acid was found to be the most effective pickling solution for AZ31 alloys. The high performance of acetic acid in improving the corrosion resistance of the substrate was mainly attributed to its high capability in reducing the Fe content on the surface. Considerable enhancement in the corrosion protection properties of an AZ31 magnesium alloy after acid pickling in the acetic acid solution has also been reported in other works [68,69]. Importantly, the use of stand-alone acetic acid solution may result in the formation of black regions on the Mg surface, which is addressed as “smutting” in the ASM handbook [70]. The formation of black regions is reported to be intensified at higher concentrations of acetic acid [71]. Therefore, as suggested in the ASM handbook, and followed in some related scientific works [71,72,73], nitrate salts were added to the acetic acid pickling solution, which reduces the substrate removal rate and formation of black regions during the pickling step. 

Cleaning of the surface by the removal of the undesired contaminants and phases is not the only goal of acid pickling. A thin layer is usually formed on the magnesium surface as the reaction of the magnesium alloy components with the acid species. Therefore, this thin layer can be considered as a conversion coating (see Section 3). The formation of this layer can be advantageous in many ways. First, as it usually has a degree of surface protection, it can be used to control the etching rate during the pickling. Second, the formation of this layer can mitigate the high reactivity of the magnesium surface, preparing it for the next step during the coating process. This is one of the essential requirements in the electrochemical plating method (e.g., [74,75,76]). Third, the formation of this layer provides a relatively homogeneous substrate for the subsequent coating, especially for alloys with electrochemical heterogeneities on the surface such as AZ91. 

In the following part, the most common acid pickling compositions and their effects on the magnesium alloys are reviewed. Moreover, an overview of the literature focusing on the effects of different acid pickling solutions on the surface properties of magnesium alloys is provided in Table 1.

**Mixture of nitric and chromic acids, H_2_CrO_4_ + HNO_3_**

Mixture of nitric acid (HNO_3_) and chromic acid (H_2_CrO_4_) is a long-known solution with excellent pickling properties that has been used for a variety of metals in various industries. Immersion in this acidic solution removes the superficial oxide/hydroxide layer on magnesium alloys and forms a new homogeneous composite layer made of chromium and magnesium oxide/hydroxide [77,78]. This composite layer is highly passivating and hinders further etching of the magnesium substrate. Therefore, a certain concentration of HNO_3_ is usually added to the chromic acid bath to accelerate the dissolution rate of magnesium [77,79]. The thin deposited composite layer after pickling in a chromic acid solution can promote the nucleation of subsequent chromium(VI) conversion coating [80]. The thin deposited layer itself is also sometimes considered as the chromate conversion coating compared to the newly developed conversion coatings [81]. Notorious for its high toxicity and carcinogenic effect, chromium (Cr(VI)) is being phased out in most industries.

**Phosphoric acid, H_3_PO_4_**

**Figure 5:**
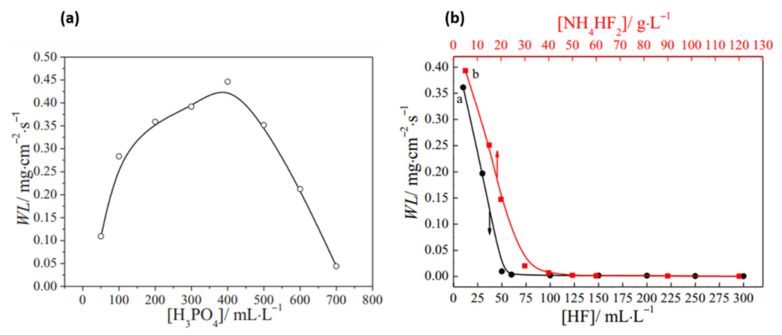
(**a**) The weight loss of an AZ31 Mg alloy immersed in H_3_PO_4_ solution with different concentrations for 60 s [83]. (**b**) The weight loss of an AZ31 Mg alloy in aqueous solutions containing 400 mL/L H_3_PO_4_ and different concentrations of HF/NH_4_HF_2_ [83]. Reprinted from [83] with permission from IOP, respectively.

When immersing magnesium plates in phosphoric acid, local alkalinization due to the cathodic reaction leads to deprotonation of the phosphoric acid molecules to PO_4_^3−^, HPO_4_^2−^, and H_2_PO_4_^−^, depending on the local and bulk pH. Therefore, a thin film, mainly composed of insoluble Mg_3_(PO_4_)_2_ and MgHPO_4_.3H_2_O, precipitates on the magnesium surface [82]. Figure 5a represents the weight loss of an AZ31 magnesium alloy in phosphoric acid solution with different concentrations. The etching rate in phosphoric acid solution increases with concentration up to a point where the deposition of Mg_3_(PO_4_)_2_ (along with AlPO_4_ and Zn_3_(PO_4_)_2_ in the case of AZXX alloys) on the surface overcomes the magnesium etching [77,83,84]. Although this semi-compact compound layer can help in controlling the etching rate, it can pose an adverse effect on the adhesion between the substrate and the subsequent coating, if it surpasses the optimal thickness [83].

Improved etching performance of the phosphoric acid can be achieved by adding other components into the solution. The addition of HNO_3_ was reported to increase the etching rate, but with the formation of a less compact layer with many active sites on the magnesium surface [77]. The addition of Na_2_MoO_4_ to the phosphoric acid pickling solution has an inhibition effect against the rapid substrate etching. Precipitation of molybdenum oxides/hydroxide on the magnesium surface results in the suppression of both cathodic and anodic activities [84]. 

One of the drawbacks of using H_3_PO_4_ is that the formation of the phosphate layer leads to a dark grey surface appearance compared to the shiny metallic surface treated by nitric or sulfuric acids [61,85].

**Hydrofluoric acid, HF**

The etching rate of the hydrofluoric acid pickling solution has been reported to be significantly less than that of nitric acid [86] and phosphoric acid [85,86] with the same concentration. This is due to the instantaneous formation of a protective passive film of MgF_2_, MgF_2-x_OH_x_·yH_2_O, or a mixture of Mg(OH)_2_ and MgF_2_ on the Mg surface [87,88]. The formed MgF_2_ layer covers the surface of the magnesium alloys with high homogeneity, which in turn, leads to the uniformity of subsequent coatings. Interestingly, preferential dissolution of some second phases such as Mg_17_Al_12_ in AZ91 [86,89,90], Mg_12_(Nd,Y) in WE54 [91], and Al_x_Mn_y_ in AZ31 [92] after exposure of the magnesium alloys to HF solutions has been observed. This might be attributed to the high reactivity of the alloying element, specifically aluminum, to HF. As a result, the electrochemical inhomogeneity of the Mg substrate itself is also mitigated.

Figure 5b represents the weight loss of the AZ31 magnesium sample after immersion in 400 mL/L H_3_PO_4_ with different concentrations of HF. It can be seen that weight loss decreases rapidly to values near zero with an increasing concentration of HF due to the strong passivation properties of the formed MgF_2_ [83,93]. Therefore, the stand-alone HF is not usually employed to remove surface impurities or modify the surface roughness, but rather to modify the surface electrochemical activity in preparation for the subsequent coating process. For instance, HF pretreatment is often used prior to Ni–P electroless plating on magnesium to provide a protective and less active MgF_2_ film on the magnesium surface as it is essential to mitigate the high corrosion rate of magnesium alloys during Ni–P plating [83,94,95,96]. 

In light of reducing the use of a highly toxic and volatile HF solution, the use of less volatile and easier to handle NH_4_HF_2_ as a source of fluoride ions has been investigated [18,83,97,98]. The effect of the concentration of NH_4_HF_2_ on the etching rate of the Mg substrate is shown to be similar to that of the HF solution due to the formation of a passive MgF_2_ film (Figure 5b).

Concentrated HF solutions can also be used for conversion coating applications to provide temporary corrosion protection. In this case, compared to the HF pickling procedures, a relatively extended treatment time, ranging from several hours [55,99,100,101] to several days [92], was investigated in order to achieve higher corrosion protection performance. More details about the magnesium fluoride-based conversion coating is provided in Section 3.

**Nitric acid, HNO_3_**

HNO_3_ etches the surface without forming a precipitation layer with protective properties on the surface. The etching rate of magnesium alloys increases with the concentration of HNO_3_ in the etching solution due to supplying more H^+^ and NO_3_^−^. H^+^ maintains the acidity of the solutions that tend to shift to an alkaline condition due to the cathodic reaction of H_2_ evolution. Furthermore, in acidic conditions, the nitrate ion can be electrochemically reduced and facilitates the magnesium dissolution [77,102]. The higher convection due to the higher H_2_ generation and the local temperature increase leads to higher etching rates [103].

Although the reaction of Mg with HNO_3_ does not result in any insoluble products that precipitate on the Mg surface, the high alkalinity in the proximity of the Mg surface still leads to the formation of a thin layer of Mg(OH)_2_ [104]. Such a layer is reported to be a few hundreds of nm after 20 s immersion of AZ91D in a 0.5 wt.% HNO_3_ [86].

**Organic acids**

In spite of the diverse variety of organic acids, only a minimal number of them have been investigated as the main component of pickling solutions. Among all the organic acids, acetic acid has been studied and used considerably more so than other organic acids [62,69,71,72,105,106], and even the mixture of 115–300 g/L acetic acid + 30–75 g/L NaNO_3_ has been offered as a suitable pickling solution for wrought magnesium alloys in standards and handbooks [107,108]. Knowing the significant importance of the acid pickling step toward the improvement in the corrosion protection properties of bare and coated Mg alloys, the currently available knowledge about the use of organic acids as the pickling solutions is notably scant; and further research on the potential organic acids in this regard will generate enormous added value for corrosion protection technologies. For instance, using the acids of complexing agents for the acid pickling step is of great promise after their recently discovered inhibition effect against the corrosion of Mg alloys [109,110,111].

#### 2.3.3. Case Study: AZ91 Magnesium Alloy

Apart from providing a clean, scale-free, rough enough surface with desired surface activity, acid etching must provide a surface with a uniform chemical composition in order to avoid any discontinuity in the subsequent coating. The chemical composition uniformity of the substrate is even more important when the subsequent coating is formed through a reaction with the substrate (e.g., conversion coating). In the case of multi-phase alloys such as the commercially common AZ91 alloy, achieving a uniform surface after the etching step is a serious challenge. The AZ91 alloy contains two main phases: aa magnesium-rich α phase and an Al-rich β phase (Mg_17_Al_12_). The electrochemical potential difference between the two phases causes the β phase to act as a cathode against the anodic α phase [112,113]. Therefore, during immersion in an acid pickling solution, microgalvanic corrosion occurs between these two phases. Due to the different local conditions (pH and ion concentration), the chemistry of the deposited film is different in each of these phases.

**Figure 6:**
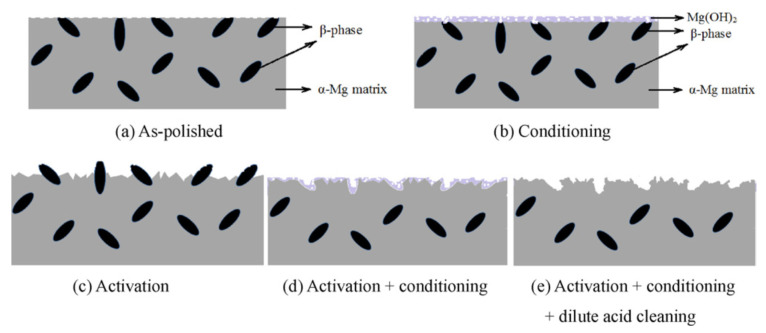
Schematic representation of the surface after the various pre-treatment processes. “Activation” refers to acid pickling, “conditioning” implies alkaline treatment. Reprinted from [52] with permission from Elsevier.

In acid pickling, the α phase is attacked by the acid, and the β phase remains almost cathodically immune. Therefore, the β phase becomes more protruded during etching, which leads to a rougher surface over time. Removal of the α-Mg phase continues until the mechanically unstable protruded β phase is detached from the surface, and more α phase is revealed underneath. The detachment of the β phase and etching of the α phase is repeated continuously during the acid pickling. Therefore, there is always a fraction of the β phase present on the surface, keeping the surface compositionally heterogeneous. In order to overcome this inherent heterogeneity of the substrate, one effective approach is the selective removal of all of the β phase, the minor phase, from the surface to reach a layer made up of a single α phase. The Al-rich β phase can be preferentially etched using highly alkaline solutions. Figure 6 shows the schematic representation of a dual-phase Mg–Al alloy surface morphology after different pre-treatment processes. In this picture, “activation” refers to acid pickling treatment, and “conditioning” refers to the alkaline treatment of the β phase removal [52]. The reaction of the β phase in the AZ91 matrix with an alkaline solution is a kinetically slow process. The constant formation of magnesium hydroxide covers the entire surface and impedes the reaction of alkaline solution with the β phase (see Figure 6b). Yang et al. [52] found out that a pre-treatment process with a sequence of 1—activation, 2—conditioning, and 3—dilute acid cleaning (Figure 6c–e) can provide an active surface with a low fraction of β phase. The obtained electrochemical homogenous surface is beneficial to achieving a uniform subsequent cerium-based conversion coating [114] or Ni-electroplating [52,115] on an AZ91D magnesium alloy.

**Figure 7:**
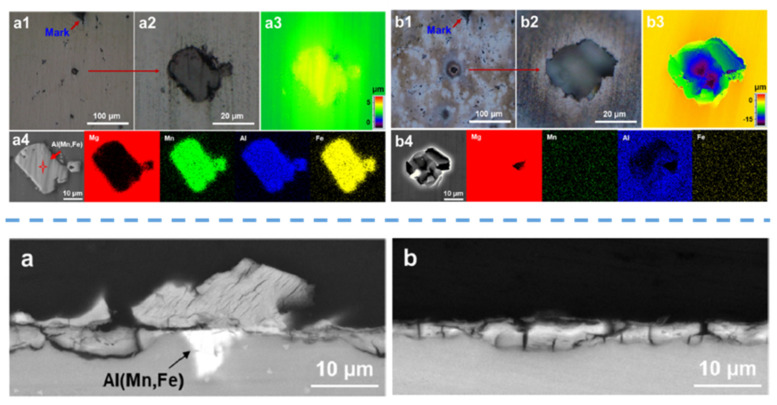
(**Top**) Surface morphology of Mg alloy AZ91 and the EDS results of the Al_x_(Mn,Fe)_y_ phase (**a1**–**a4**) before and (**b1**–**b4**) after the pre-treatment. (**Bottom**) Electron probe microanalysis technique (EPMA) mapping of the cross-section of phosphate conversion coating on the (**a**) untreated and (**b**) pre-treated AZ91. Reprinted from [110] with permission from Elsevier.

In a recent work by Liao et al. [110], a novel pre-treatment chemistry was designed to specifically target the Al_x_(Mn,Fe)_y_ phases as impurities in the AZ91 alloys. The proposed pre-treatment bath is in an alkaline condition to avoid the severe etching of the α-Mg phase. The addition of ethylenediaminetetraacetic acid (EDTA) into the pre-treatment bath lowers the reduction potential of Fe and Mn in the Al_x_(Mn,Fe)_y_ phases. EDTA is able to form a soluble complex with Mg^2+^, which is stable even at highly alkaline conditions of the pre-treatment bath [116]. Furthermore, the addition of the strong oxidizing agent NO_3_^−^ assists in the dissolution of the second phases. Figure 7 (top) shows an example of the corresponding successful removal of Al(Mn,Fe) impurities. Consequently, a more uniform subsequent phosphate conversion coating was achieved (Figure 7 (bottom)), which in turn, results in superior corrosion resistance.

**Table 1:** Overview of the selected pre-treatment procedures reported in the literature.

Pre-Treatment	Concentration/ Other Parameters	Substrate	Subsequent Coating	Effects on Properties of the Substrate or the Coating	Reference/Year
-acid pickling: 1-H_3_PO_4_ 2-HF 3-HNO_3_	Duration: 60–600 s 85% 50% 70%	AM50 and AZX310	-	Among the tested acid solution, HNO_3_ exhibited the most effective result to reduce the corrosion rate in 3.5 wt.% NaCl.	[85] 2017
-acid pickling: 1-CH_3_COOH + Ca(NO_3_)_2_ 2-C_2_H_2_O_4_.2H_2_O 3-C_6_H_8_O_7_	Duration: 15–120 s 100–200 g/L + 50 g/L 20 g/L 40–120 g/L	AZ31	-	4 µm etching is claimed to be sufficient to ensure the reduction of Fe impurity level close to that of bulk. Acetic acid-based solution showed the best result in terms of impurity removal.	[62] 2010
-acid pickling: 1-H_2_SO_4_ 2-HNO_3_ 3-H_3_PO_4_	Duration: 15–120 s 10–50 g/L 20–80 g/L 40–80 g/L	AZ31	-	5 µm etching is claimed to be sufficient to ensure the reduction of Fe impurity level below 100 ppm. Nitric acid had the best performance to reduce the corrosion rate of bare AZ31.	[61] 2010
-alkaline cleaning: NaOH + Na_3_PO_4_.12H_2_O + NaSiO_3_.10H_2_O + OP-10 -acid pickling: H_3_PO_4_ (85% V/V)+ Na_2_MoO_4_.2H_2_O -activation: NH_4_HF_2_	Duration: 8–10 min 40 g/L 20 g/L 20 g/L 3 mL/L Duration: 5–10 s 200 mL/L 1–20 g/L Duration: 6–10 min 200 g/L	AZ91D	Electroless Ni–P plating	Reduction in etching rate with increase in the concentration of Na_2_MoO_4_.2H_2_O. Increase in Ni–P plating rate with increase in concentration of Na_2_MoO_4_ from 0.5 g/L to 7 g/L.	[84] 2011
-acid pickling: 1-HF 2-HCl 3-HNO_3_	Duration: 20 s 0.5 and 11 wt.% 0.5 wt.% 0.5 wt.%	AZ91D	Stannate conversion coating	Best corrosion protection performance in 0.05 M NaCl in the case of HF compared to other acid pickling solutions.	[86] 2011
-acid pickling: 1-H_3_PO_4_ 85% 2-HCl 37% 3-HNO_3_ 68% 4-C_6_H_8_O_7_ -conditioning: NaOH	200 mL/L, 30 s 5 mL/L, 30 s 30 mL/L, 30 s 20 g/L, 45 s 200 g/L, 65 °C, 30 min	AZ91D	Zn immersion coating	H_3_PO_4_ and HNO_3_ pickling solutions preferentially attacked the β phase/matrix interface. Combination of acid pickling + conditioning treatment can provide an electrochemically uniform substrate, which results in a uniform subsequent Zn immersion coating.	[52] 2012
-acid pickling: H_3_PO_4_ -activation: 1-HF 2-NH_4_HF_2_	Duration: 1 min 50~700 mL/L Duration: 8 min 10–300 mL/L 5–150 mL/L	AZ31	Electroless Ni–P plating	The highest etching rate of H_3_PO_4_ was achieved at 400 mL/L concentration. Pickling with H_3_PO_4_ improve the corrosion resistance of the subsequent electroless Ni–P plating when the concentration is less than 400 mL/L. The best corrosion resistance performance obtained when pickling with H_3_PO_4_ and subsequent NH_4_HF_2_ activation were performed.	[83] 2014
-acid pickling: 1-hydrofluoric acid 2-acetic acid 3-N_3_PO_4_ + NaOH	10% *v*/*v*, 10min 0.05 M, 30 s 10 g/L + 50 g/L, 40 min	AZ91	Sol–gel (TEOS/MTMS)	Na_3_PO_4_ + NaOH pre-treatment offers a better surface condition for the subsequent sol–gel deposition as compared to the acid pickling pre-treatments, which, in turn, leads to a more corrosion protective sol–gel coating.	[54] 2019
1-sand blasting 2-grinding 3-polishing	-corundum particles (180 µm) -emery paper #150, 400 and 1000 −2.5 µm alumina slurry	AZ91	Phosphate conversion coating	Lower surface roughness resulted in a more uniform and denser coating. Different coating composition was observed at the valleys and peaks of the rough surface treated by grinding.	[13] 2019
-acid pickling: 1-HNO_3_	Duration: 90 s 1 M	AZ31	Polycaprolactone (PCL) electrospinning	Pre-treatment with HNO_3_ significantly reduce the corrosion rate of the bare AZ31 and PCL-coated samples in SBF solution.	[117] 2016
-acid Pickling: 1-HNO_3_ 2-H_3_PO_4_	Duration: 30–180 s 1 M 1 M	Mg0.6Ca	CaP conversion coating (as the result of immersion in SBF)	Higher deposition rate of CaP phase in SBF after acid pickling. Lower corrosion rate in SBF after acid pickling. Slightly higher corrosion resistance of the substrate treated by HNO_3_ compared to that treated by H_3_PO_4_	[118] 2021
-pre-treatment NO^−^_3_ + EDTA	Duration: 30 min Temperature: 60 °C 0.1 M PH: ~13.5	AZ91	Phosphate conversion coting	The successful dissolution of Al_x_(Mn,Fe)_y_ impurity phase and formation of electrochemically uniform surface. Formation of more uniform phosphate conversion coating with superior corrosion protection properties.	[110] 2022

## 3. Conversion Coatings

### 3.1. Introduction

Chemical conversion treatments are relatively effective, simple, and cheap methods, which are widely used in industrial applications for short-term temporary protection and providing adhesion for paints to Mg alloy surfaces. Until recently, chromate containing conversion products remained the most efficient conversion solution for Mg alloys and a number of commercial products have explored advantageous properties of Cr(VI) as a corrosion inhibitor. Cr(VI)-free alternatives are based on different major components that include:

- Phosphate with permanganate;
- Metal phosphates (Ca^2+^, Zn^2+^, Mn^2+^, Sr^2+^);
- Rare earth (Ce^3+/4+^), La^3+^, Y^3+^);
- Permanganate with vanadate/molybdate/wolframate/zirconate;
- Permanganate with HF;
- Fluorides;
- Hexafluorozirconate, hexafluorotitanate, and other fluorometallates;
- Stannates;
- Phytates and other organic polymers;
- Al–Mg layered double hydroxides;
- Cr(III)-less favorable option owing to generation of Cr(VI) during exploitation.

**Figure 8:**
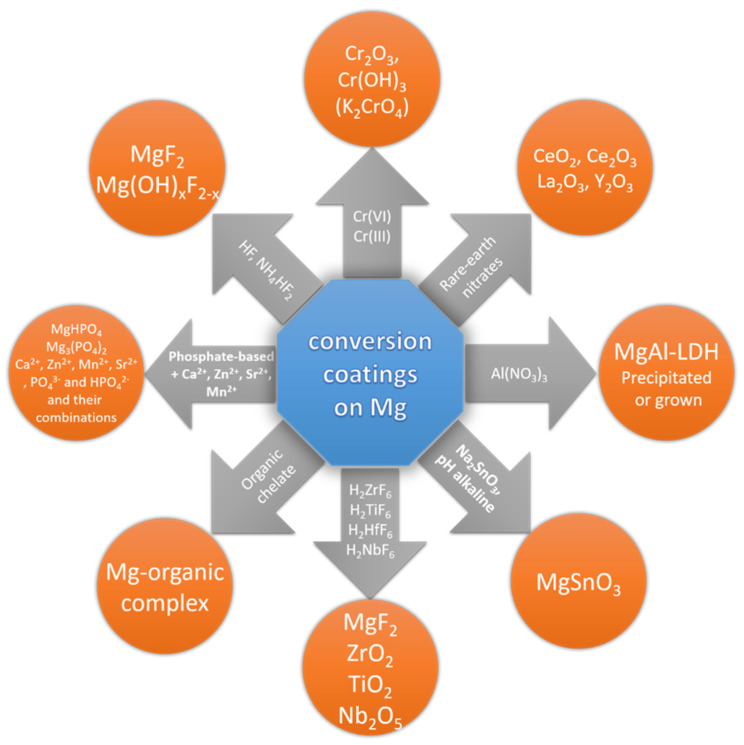
General overview of the most frequently used conversion treatments and corresponding products formed on the magnesium surface.

Most of the conversion coating compositions are experimental and non-commercial, and some are proprietary, but the general chemical compositions and basic mechanisms for all can be found in book chapters [119,120,121,122], several review papers [122,123,124], and multiple original research papers accounting roughly to 950 items indexed by the Scopus research database and up to ca. 100 patents/patent applications, as for the beginning of 2022. An overview of the selected conversion formulations and coatings is presented in Figure 8 and Table 2 at the end of this section.

**Table 2:** The overall electrochemical and chemical reactions in the titanate conversion bath and at the surface of the AZ31 substrate. Reprinted from [125] with permission from IOP. The conversion bath was composed of 0.01 M TiCl_4_, 0.01 M H_2_SiF_6_, and 5 mL/L HNO_3_ at 40 °C at pH 4.

H^+^ dissociation	H_2_SiF_6_ ⇄ 2H^+^ + SiF_6_^2−^	(1)
SiF_6_^2−^ dissociation	SiF_6_^2−^ + 4H_2_O ⇄ Si(OH)_4(s)_ + 4H^+^ + 6F^−^	(2)
TiCl^4^ dissociation	TiCl_4_ + 4H_2_O ⇄ Ti(OH)_4(s)_ + 4HCl	(3)
Reaction of TiO_2_ and F^−^	TiF_6_^2−^ + 2H_2_O ⇄ Ti(OH)_4(s)_ + 4H^+^ + 6F^−^	(4)
Magnesium dissolution	Mg → Mg^2+^ + 2e^−^	(5)
Aluminum dissolution	Al → Al^3+^ + 3e^−^	(6)
Proton reduction	2H^+^ + 2e^−^ → H_2(g)_	(7)
Hydroxide formation	Mg^2+^ + 2OH^−^ → Mg(OH)_2(s)_	(8)
Reaction of Mg^2+^ and F^−^	Mg^2+^ + 2F^−^ → MgF_2(s)_	(9)
Hydroxide formation	Al^3+^ + 3OH^−^ → Al(OH)_3(s)_	(10)
Reaction of Al^3+^ and F^−^	Al^3+^ + 3F^−^ → AlF_3(s)_	(11)
Reaction with more F^−^	Al^3+^ + 6F^−^ → AlF_6_^3−^_(aq)_ or/and AlF_3(s)_ + 3F^−^ → AlF_6_^3−^_(aq)_	(12)
Mg(OH)_2_ dehydration	Mg(OH)_2(s)_ → MgO_(s)_ + H_2_O	(13)
Al(OH)_3_ dehydration	Al(OH)_3(s)_ → Al_2_O_3(s)_ + H_2_O	(14)
Ti(OH)_4_ dehydration	Ti(OH)_4(s)_ → TiO_2_ + 2H_2_O	(15)
Si(OH)_4_ dehydration	Si(H)_3(s)_ → SiI_2(s)_ + 2H_2_O	(16)

Chromate-based conversion layers have been among the oldest and most efficient approaches for Mg treatments for decades in a similar way as for other metallic substrates. However, in-depth investigations of conversion layer formation and corrosion protection mechanisms are mostly limited to aluminum alloys and steel, which are also commonly assumed to apply to magnesium alloys [1]. Indeed, determining the mechanism of chromate conversion coatings on Mg alloys is an important step toward finding a replacement with the desired protection characteristics. In one of the few investigations on Mg alloys, Pommiers et al. [80] detailed the step by step formation of a chromate conversion coating (CCC) on a Mg alloy (EV31A). 

The predominant species of the CCC bath at the common pH (between 1 and 3) and Cr(VI) concentration (around 50 mM) is dichromate (Cr_2_O_7_^2−^), which oxidizes the Mg substrate and generates Cr(III) species. Cr(III) species then precipitate in the form of Cr(OH)_3_ and Cr_2_O_3_ as the main components of the conversion layer [80]. It is also believed that an inorganic polymer of Cr(OH)_3_ can be formed through a chemical condensation reaction [1]. In addition, during the conversion coating formation, Cr(VI) species can be entrapped within the conversion film, which are identified in the form of CrO_3_/K_2_CrO_4_ and is observed as a precipitate with an orthorhombic crystallographic structure [80]. They are believed to be the main responsible for the unique self-healing properties of CCCs. Cr(VI) species can be released from the coating into a corrosive aqueous medium and reach a corroding substrate at a defected region of the coating. The subsequent reduction to insoluble Cr(III) oxide/hydroxide heals the active defect.

The CCC is the reference for the protection of magnesium alloys, but it urgently requires efficient substitutes due to its carcinogenicity. As above-mentioned, its mechanism of protection shows that the species responsible for its barrier properties is trivalent chromium oxide Cr_2_O_3_ [123]. Naturally, Cr(III)-based conversion treatments have been developed and studied on steel, zinc, and aluminum [126,127,128,129,130,131,132]. The reports on the application of Cr(III)CC on magnesium are scarce [133,134,135] and mostly limited to commercial products that contain several more additives to the conversion coating bath. Additionally, the patents that typically claim the applicability of specific Cr(III) formulations to a number of metallic surfaces including Mg do not disclose the details of electrochemical or corrosion performance [136]. Although the environmental amiability of Cr(III) remains a matter of detailed study, one of the important advantages of Cr(III)-containing coatings is that they are resistant to thermal shocks, while Cr(VI)CC layers lose their corrosion resistance if heated above 70 °C [123,137].

### 3.2. Phosphate-Based Conversion Coatings (PCC)

Phosphate-based conversion coatings (PCC) are one of the most commercially used alternatives to chromate conversion coatings on steel and aluminum [138,139,140,141]. The commercial use of PCC for Mg alloy substrates is still rather limited but is gaining ground. The investigation of corrosion protection properties of phosphate-based conversion coatings on Mg alloys remains a highly active R&D topic. A recent review has compiled the bath composition and deposition mechanisms of multiple magnesium phosphatization approaches [142]. For other metallic substrates, PCC is broadly used in almost all industry fields, mainly in automotive assembly, coil coating, and metal surface protection. In the aerospace industry, phosphatization is mainly used in combination with Cr-based systems. It is cost-effective, with good corrosion resistance and good adhesion to paints. Accurate operation and constant control of bath analytics are required to achieve the best and most reproducible performance. Moreover, PCC is usually a multi-step process that typically includes an activation step (for the formation of seed crystals on the substrate surface). The main components of the phosphate-based conversion process (phosphoric acid, sodium, potassium, ammonium, calcium, strontium, zinc, and manganese (di)hydro-phosphates) do not appear in the European environmental restrictive lists of “Substances restricted under REACH”, “Authorization List”, “Candidate List of substances of very high concern for Authorization”, and “Submitted recommendations” [143]. On the other hand, the European Commission supported the ban on phosphates in consumer detergents in 2011 [144]. This is due to the fact that an excessive amount of phosphates stimulate algae growth at the expense of other aquatic life. Thus, plants operating phosphate conversion coatings are subject to strict waste water cleaning regulations.

Phosphate solutions are usually modified by adding cations: magnesium, zinc, calcium, strontium, manganese and accompanying anions: nitrate, nitrite, fluoride, silicate, molybdate, vanadate; organic salts, and combinations of them to tune the coating properties according to their applications. For instance, the addition of Ca^2+^ in phosphate baths not only improves the corrosion protection properties of the formed coating, but also causes the deposition of biocompatible compounds [142,145,146,147].

**Figure 9:**
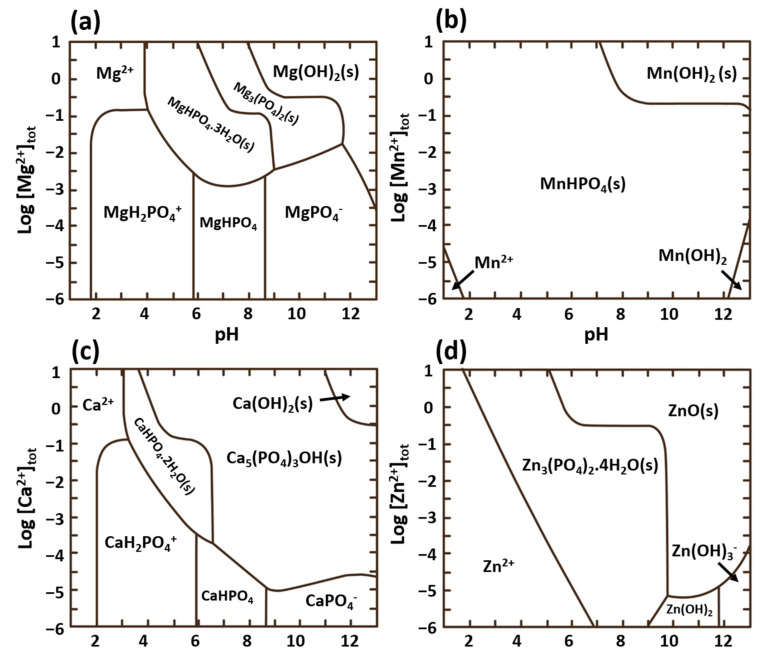
Predominance area diagrams for solutions containing 0.1 M phosphate as the function of concentrations of (**a**) Mg^2+^, (**b**) Mn^2+^, (**c**) Ca^2+^, and (**d**) Zn^2+^ ions and solution pH. Hydra-Medusa software was used to simulate the predominance area. This figure is inspired by [124].

Figure 9 illustrates how the variations in pH and concentration of commonly investigated Mg^2+^, Mn^2+^, Zn^2+^, and Ca^2+^ in a phosphate bath result in different thermodynamically stable phases. For instance, it can be observed that Mn^2+^ can form an insoluble phosphate that is stable in a wide range of pH and Mn^2+^ concentrations. However, when Ca^2+^ is added to the phosphate conversion coating bath, two phases of Ca_5_(PO_4_)_3_OH (hydroxyapatite, HA) and CaHPO_4_.2H_2_O (brushite, dicalcium phosphate dihydrate, DCPD) can be the predominant compositions of the formed film, depending on the pH and Ca/P ratio in the bath [124,147,148].

Ca–P–Mg containing degradation products naturally form on the surface of bioabsorbable Mg implants [149]. Ca-PCCs on Mg alloys possess high biocompatibility and have been extensively studied in bio-applications [150,151,152]. Along with the main phases of HA and DCPD, octacalcium phosphate (OCP) [153,154] and the minor presence of Ca_3_(PO_4_)_2_ have also been reported [155,156,157,158,159] that can be transformed to HA via a post-immersion in 1 M NaOH solution at elevated 80 °C [160]. The alkali post-treatment also promotes the transformation of the DCPD to HA [161,162], evident from the thermodynamic illustration of the stable phases in Figure 9, which in turn can significantly enhance the corrosion resistance of the coating [162,163]. The alkaline post-treatment solution often contains Ca^2+^ ions to further promote the formation of HA [162,164]. In this case, the addition of chelating agents such as EDTA can prevent the premature precipitation of calcium hydroxide in the solution [162]. 

The increase in local alkalinity due to the corrosion of Mg also leads to the instability of DCPD and its transformation into HA. Thus, some reports have claimed the self-healing properties of DCPD coatings via the re-precipitation of DCPD on the defected region in the form of HA and amorphous calcium phosphate compounds [165,166]. The presence of Mg^2+^ ions may impede the crystallization of HA [167,168], and instead, amorphous calcium phosphate phases could form [165]. 

The re-precipitation of calcium phosphate compounds from HA has also been reported [169]. However, considering the lower solubility of HA compared to DCPD, a stronger self-healing is expected from the DCPD coating [165], disregarding the overall corrosion protection of DCPD compared to that of HA.

**Figure 10:**
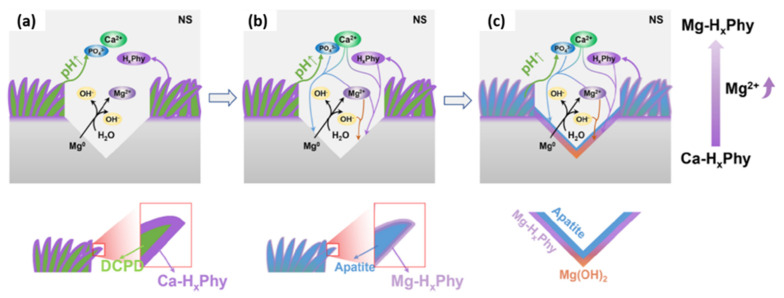
Schematic diagram of self-healing mechanism of the scratched phytate-modified DCPD coatings in 0.9 wt.% NaCl solution (NS) (DCPD: dicalcium phosphate dihydrate coating; Phy: phytate). The self-healing mechanism is in order from (**a**–**c**). Reprinted from [170] with permission from Elsevier.

It has been reported that a post-treatment of a DCPD coating in a phytic acid solution can provide self-healing properties [170]. The proposed mechanism, which is illustrated schematically in Figure 10a–c, is as follows: A layer containing a complex between phytic acid and Ca^2+^ is formed on DCPD after the post-treatment. The transition of DCPD to HA during the corrosion of the Mg substrate leads to the consumption of Ca^2+^, which in turn releases the phytic acid molecule. The released phytic acid molecule re-precipitates in the form of a complex with Mg^2+^ generated on the active corrosion region.

Bear in mind that the precipitation of calcium phosphate compounds on the substrate supplied by the species from physiological media does not represent a self-healing characteristic of a coating, but rather an external effect that can occur regardless of the type of coating [165,171,172].

Zn-PCCs mainly consist of Zn_3_(PO_4_)_2_.4H_2_O (hopeite), which is precipitated by a chemical reaction between Zn^2+^ and H_2_PO_4_^−^ ions in the solution favored by increasing the solution pH [138,173,174,175,176]. Small quantities of metallic Zn and ZnO in the coating have also been reported [150,156,177]. Yuan et al. [178] showed that a hydrothermal post-treatment of a Zn-PCC on an AZ61 magnesium alloy in the same zinc phosphate solution containing stearate at 140 °C for 24 h could promote the formation of the ZnO phase in the coating. The Zn^2+^ ions are usually introduced in the bath solution by the addition of Zn(NO_3_)_2_ or ZnO. For aluminum alloys, zinc-phosphate conversion coatings have been used commercially for a long time, specifically with the purpose of providing high adhesion between the paint and the substrate [138,179]. For the Mg substrate, early studies reported an excellent adhesion between paint and a zinc-phosphate coating [180], which surpassed the adhesion between a chromate conversion coating and the same paint. However, the main focus of the applications for zinc phosphate conversion coating on Mg is on the bio-absorbable implant applications such as stents due to the basic safety of Zn in biomedical applications [147,181]. 

Sr^2+^ is another cation additive to a phosphate-based conversion coating bath that also benefits from high biocompatibility and has remarkable stimulation properties for bone formation [182,183]. Sr has a similar chemical behavior as Ca and can substitute the Ca atoms in a hydroxyapatite structure in different atomic ratios to form strontium apatite [184]. 

**Figure 11:**
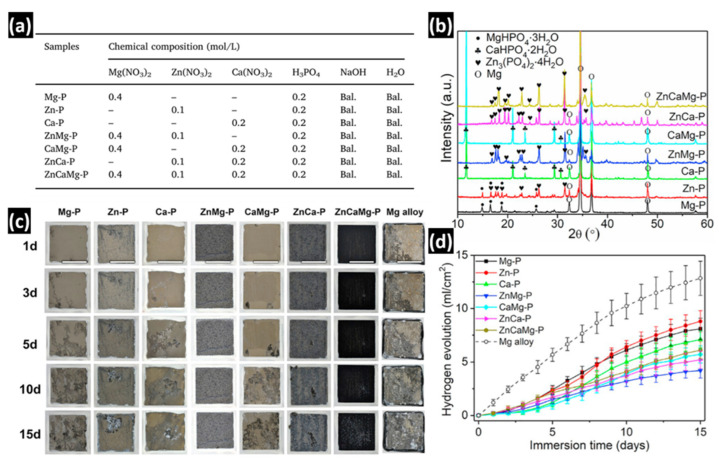
(**a**) Chemical composition of the phosphate conversion baths used in [150]. (**b**) The XRD patterns of the formed conversion coatings from the bath composition in (**a**) of AZ31. (**c**) Surface appearance and (**d**) hydrogen evolution of the AZ31 substrate coated with different phosphate conversion coating in (**a**) during immersion in Hank solution at 37 °C. Reprinted from [150] with permission from Elsevier.

Simultaneous addition of the above-mentioned cations into the bath has also been studied with the aim of improving the corrosion protection of the final formed coating. Different phosphate compounds of the cations can simultaneously precipitate depending on their thermodynamic stabilities. For instance, the change in Gibbs free energy, ΔG, of the phosphates Ca_3_(PO_4_)_2_ and Zn_3_(PO_4_)_2_ is −187.71 kJ/mol and −186.33 kJ/mol, respectively. Thus, they can simultaneously precipitate on the surface [156,177] or in a co-existing Zn/Ca phosphate of the CaZn_2_(PO_4_)_2_ phase [156,185,186] due to their similar crystallographic structure. Bear in mind that in order to predict the predominant composition of the formed PCC, it is necessary to consider the stability of these phases with respect to other possible phases. For instance, as previously stated and illustrated in Figure 9, the formation of brushite and HA is thermodynamically favorable when compared to Ca_3_(PO_4_)_2_. Zai et al. [150] carried out a comparative study on different combinations of additional cations, namely, Mg^2+^, Ca^2+^, and Zn^2+^ in a phosphate conversion bath with a constant concentration of each cation and controlled pH of 2.7 on an AZ31 magnesium alloy. The composition of different phosphate conversion baths is shown in Figure 11a. The precipitation of Zn_3_(PO_4_)_2_ takes priority with respect to the hydrophosphates of Ca and Mg as it is the main phase that appeared in the X-ray diffraction (XRD) pattern of the conversion coatings when Zn was present in the conversion bath Figure 11b. This can be attributed to the lowest solubility of Zn_3_(PO_4_)_2_ (K_sp_ = 9 × 10^−33^) compared to those of MgHPO_4_ (K_sp_ = 1.5 × 10^−6^) and CaHPO_4_ (K_sp_ = 1.0 × 10^−7^). Among all of the tested combinations of phosphate coatings on AZ31, the phosphate conversion coating obtained from the bath containing Mg and Zn ions showed the lowest corrosion rate and the least change in the sample appearance during immersion in Hank solution. 

The thermodynamic stability of possible phases in the presence of more than one cation can be taken as the basis of the prediction of the conversion coating composition. Hou et al. illustrated that in a PCC bath containing both Mg^2+^ and Mn^2+^ ions, the concentration of Mg^2+^ can have a dual effect on the precipitation of MnHPO_4_. The formed MgHPO_4_ that precipitates during the conversion coating can act as the nuclei for the precipitation of MnHPO_4_. Thus, an increase in Mg^2+^ leads to a higher precipitation rate of MnHPO_4_, which helps to form a coating with a higher corrosion protection performance. However, an excessive concentration of Mg^2+^ in the bath causes a reduction in the electrochemical activity of the Mg surface, which is necessary for the conversion coating growth. 

Permanganate (MnO^4−^) salts are usually added to phosphate baths in order to accelerate the phosphate treatment on magnesium alloys [187,188]. However, a higher concentration of permanganate in the solution promotes the formation of magnesium oxide film on the magnesium substrate, which retards the further dissolution of the magnesium matrix. Therefore, the higher the concentration of permanganate in the conversion bath, the thinner the final conversion coating [189]. Moreover, different manganese oxides (MnO_2_ and Mn_2_O_3_) can co-precipitate with the phosphate film [189,190,191]. Permanganate is not completely stable due to its tendency of decomposition, which causes oxygen evolution and the formation of manganese ions (Mn^2+^) in acidic media. The decomposition rate is generally slow in dilute acid solution but can be accelerated by light, heat, acidic conditions, and the presence of manganese(IV) oxide [189]. In addition to permanganate salts, other accelerating agents including NO_3_^−^ [175,180], NO_2_^−^ [175,180], and sodium dodecyl sulfate (SDS) [192,193] have been used to promote the dissolution of Mg during the conversion coating formation. 

As a general observation, phosphate conversion treatments (as well as many other conversion coating treatments for magnesium alloys) feature a coating rich with cracks that weakens the corrosion protection properties. These cracks have usually been associated with the post-immersion drying step. Shrinkage in volume due to the dehydration of compounds in the coating induces tensile stresses, leading to the formation of cracks in the coating [194,195]. Therefore, the post drying conditions can dramatically change the distribution, size, and depth of cracks. Moreover, the effect of vacuuming during surface characterization with SEM cannot be ignored [196]. In a recent study by Zhou et al. [197], in the phosphate conversion treatment on magnesium alloys with a bath modified with Ca^2+^ and VO_3_^−^ ions, some cracks were stopped in the middle of the coating thickness, suggesting that they originated from the metal/coating interface rather than from the surface of the coating. Furthermore, a “*vena contracta*” phenomenon was associated with the cause of the shape of cracks that had passed through the thickness of the coating. The cracks were wide at the metal/coating interface, necked in the middle, and regained their large width close the coating surface. However, the proportionality of the tensile stress during the dehydration process to the thickness of the coating usually led to the formation of cracks that widened from the substrate to the surface. Therefore, a hydrogen-induced cracking mechanism was proposed that is mainly caused by the coalescence of the entrapped H_2_ bubbles during the conversion coating formation.

Liao et al. [198] proposed a conversion coating bath design that could lead to a protective thick crack-free conversion coating. In their approach, the conversion coating should consist of two layers, spontaneously forming on the surface but with a decoupled growth. The inner layer is cracked, allowing for continuous substrate dissolution during the process and inducing a thick and crack-free outer layer. This two-layer coating can be achieved by implementing two categories of species in the bath, contributing to the formation of each of two inner and outer layers. The crack formation on the outer layer is mitigated by the increase in its nucleation rate, which in turn facilitates the release of local stress through the high surface area of the grain boundaries. This approach to a crack-free conversion coating was illustrated for a Mn-phosphate conversion coating on an AZ91 substrate. The cracked inner layer was made of MgHPO_4_, and the outer layer was formed by the precipitation of MnHPO_4_. The nucleation rate of MnHPO_4_ was tailored via the addition of different species in the bath including complexing agent (EDTA), NH_4_^+^, and NO_3_^−^. As a result, a 3 µm thick conversion coating with a dense MnHPO_4_ outer layer was formed, which led to a significant reduction in both the cathodic and anodic activity of the AZ91 substrate. As a result, remarkably less degradation was observed after a 120-h salt spray test (SST) compared to the bare and benchmark Mn-P coated AZ91 sample. 

Apart from the effect of the pH on the main composition of the formed coating, optimization of the phosphating bath pH is a crucial aspect of achieving the highest coating thickness. For instance, in a Ca–P conversion coating, when the bath pH is very low and the substrate dissolution rate is high, alkalization by the cathodic reaction is not sufficient to precipitate CaHPO_4_·2H_2_O locally on the Mg surface (which is the main composition of Ca-P conversion coating). On the other hand, an increase in bath pH slows down the substrate dissolution rate [145,184]. The optimized bath pH to achieve the highest coating thickness in the case of the CaP conversion coating has been reported to be in the range of 2.8–3.2 [145,148,199,200]. Such complexity on the effect of bath pH on the formation of the conversion coating has been similarly explained for other phosphate-based conversion systems such as Mg–P [201] and Zn–P [175,202].

Organic substances have been commonly added to the phosphate bath with different purposes such as reducing the amount of bath sludge (which influence the coating quality), stabilizing the phosphate bath, accelerating the Mg dissolution rate, modification of coating protective properties by forming a more compact film, and exploiting their inhibitive properties [192,203,204,205,206,207,208]. Li et al. [206] investigated the effect of the monoethanolamine (MEA) additive to a zinc phosphate bath on the morphology and the protective properties of the formed conversion coating. They observed that MEA could refine the microstructure of the coating to the most uniform and compact structure at an optimum concentration of 1.2 g/L. The concentration of 0.8 g/L was the threshold minimum for the MEA concentration to exhibit catalytic effects on the nucleation of hopeite crystals. 

**Figure 12:**
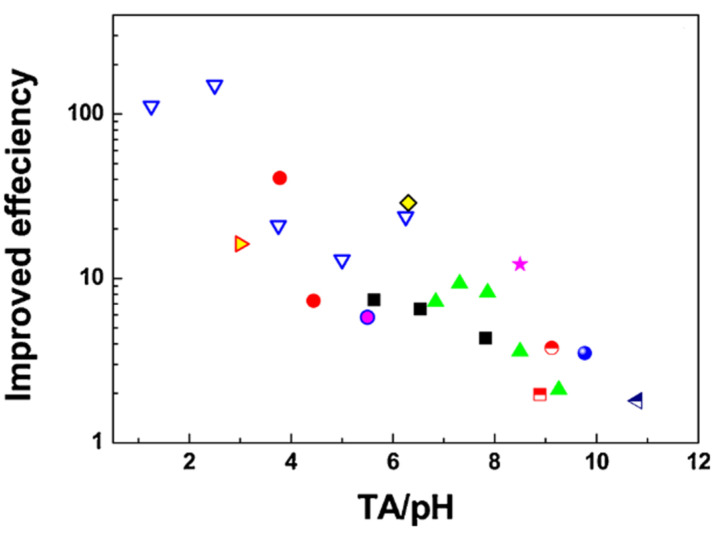
Relationships between the TA/pH and corrosion protection effectiveness of PCCs derived from the published data. Each symbol represents a specific published reference. The information of references can be found in [209], where the figure is taken from. Reprinted from [209] with permission from Elsevier.

A high number of reported PCC treatment bath recipes and conditions (pH, temperature, treatment time, testing methods, electrolytes, and tested alloys) rendered accumulated knowledge fragmentary. Wide parameter variation presented a significant hurdle for understanding the general trends important to optimize the method toward wide industrial application. Recently, it has been shown [209] that the ratio between the total, or titratable acidity (TA) and pH of the treatment bath plays a critical role in the formation of PCC with high corrosion protection. A formulation with high TA/pH contains phosphoric acid, while that with low TA/pH is composed of PO_4_^3−^ at high pH. It was shown for Mn-containing PCC that solutions with low TA/pH resulted in the precipitation of Mn–phosphate conversion layer of Mg with a homogeneous microstructure and high corrosion protection ability. This finding of the importance of low TA/pH ratio was validated by comparing the performance of various PCC (Figure 12). The concept “low TA/pH” was found to be applicable not only for Mn-PCC, but also for a wide variety of phosphating baths containing Ba^2+^, Zn^2+^, Ca^2+^, Sr^2+^, MnO_4_^−^ and F^−^ applied to the AZ, AM, ZK, and Mg–Li alloy series. 

The TA/pH ratio of a phosphate bath was taken into account in an investigation of the properties of a Mn-PCC formed on different crystallographic textures of an AZ31 magnesium alloy [210]. The lower electrochemical reactivity of the basal crystallographic orientation (0001) led to a higher TA/pH ratio compared to the other crystallographic orientations. Thus, a higher corrosion protection performance was observed on the Mn-PCC deposited on the (0001) plane. 

Obviously, the TA/pH should have a critical value below which the corrosion protection properties of the formed coating declines, simply because the limiting value of TA/pH = 0 is assigned to pure water. Moreover, the increase in pH value usually leads to the precipitation of the additional cation (e.g. Mn^2+^) in the form of hydroxide or phosphates/hydrophosphates in the conversion bath. This limitation can be solved by the addition of complexing agents that hinder the precipitation of the additional cations [198].

### 3.3. Rare Earth-Based Conversion Coatings

Rare earth (RE)-based conversion coatings (RECC) are a promising replacement for chromate conversion coatings and have been extensively studied on different metals such as steels, Al, and Mg alloys [123,124,211,212,213,214]. A recent review summarized the compositions of the coating baths and corresponding parameters, the chemistry of the deposited conversion layers, and their performance for magnesium alloys [215]. Among all of the rare earth elements, Ce is the most studied element for conversion coating treatment [215,216]. Conversion coatings based on other rare earth elements have also been reported: La^3+^, Y^3+^, Nd^3+^, Pr^3+^, Sm^3+^ and Gd^3+^ [217,218,219,220,221,222,223,224]. Their combinations have been studied with each other, other inorganic ions (Zn^2+^, Mg^2+^, Ca^2+^, Al^3+^, MoO_4_^2−^, VO_3_^−^, MnO_4_^−^, PO_4_^3−^, P_2_O_7_^4−^) [215,218,225,226,227,228,229,230,231], and organic compounds including silanes [232], gelatin [233,234,235,236], chitosan [233,234], sodium dodecyl benzene sulfonate [220], phytic acid [237,238], ascorbic acid [221], citric, and other carboxylic acids [215,220,221,233,239].

The precipitation of RE oxides/hydroxides on the substrate is the main mechanism of coating formation. These hydroxides are formed as a result of the fast rise in local pH due to cathodic reactions on the metal substrate. Competitive reactions between OH^−^ and existing metallic cations at certain environmental conditions (pH and ion concentration) determine the final composition of the precipitated film [240]. For instance, when the AZ91D magnesium alloy is immersed in yttrium nitrate Y(NO_3_)_3_ solution, there is a competition between Al^3+^,Y^3+^, and Mg^2+^ ions for OH^−^. Due to the lower solubility of Al^3+^ and Y^3+^ hydroxides, they are more favorable to deposit on the alloy surface [219]. Typically, the thickness of RECC varies within 1–5 µm.

Oxidizing agents such as NO_3_^−^ or H_2_O_2_ are usually added to the conversion bath in order to accelerate the magnesium dissolution and promote the alkalization due to the cathodic hydrogen evolution reaction [123,220,236,241,242]. Chen et al. [220] reported that the addition of 5–12 mL/L of H_2_O_2_ to the bath for cerium-based conversion treatment on the AZ31 alloy can drastically enhance the coating mass gain by about 20 times. Moreover, H_2_O_2_ can oxidize the Ce^3+^ ions in the solution to Ce^4+^ ions. Therefore, the final coating composition contains Ce(OH)_4_, Ce(OH)_3_, and Mg(OH)_2_ [243]. Cerium hydroxides are eventually dehydrated and transformed into the oxides CeO_2_ and Ce_2_O_3_ when exposed to the atmosphere [241]. The cerium oxides in the coating tend to have a more amorphous structure with the increase in H_2_O_2_ concentration in the bath [230,244]. Permanganate is another strong oxidizing agent used in Ce-based conversion baths [225,226,245]. Apart from playing a similar role in promoting the magnesium alloy dissolution, MnO_4_^−^ can be reduced and co-deposited as Mn_2_O, Mn_2_O_3_, and MnO [245,246]. Although the addition of an oxidizing agent has a positive influence on achieving a thicker conversion coating, it has been reported that if the concentration of oxidizing agents such as H_2_O_2_ exceeds a certain value, a highly porous structure is formed, which is detrimental to the protective behavior of the coating [213,221]. Achieving a thicker cerium conversion coating on the AZ91D substrate using prolonged immersion time has similarly been reported to have an adverse effect on the corrosion protection properties of the coating due to the formation of a more defective layer [236,247]. 

Post-treatments can be conducted on RECCs to improve the corrosion protection properties of the coating by achieving a denser and more homogenous coating morphology. Hydrothermal post-treatment of a cerium based CC (CeCC) in 2.5 wt.% NaH_2_PO_4_ solution at 85 °C for 5 min resulted in a decrease in the Ce(III)/Ce(IV) ratio and partial conversion of Ce-based compounds to hydrated cerium phosphates [239]. Although the thickness of the CeCC was negligibly changed, the modification in the coating chemistry and morphology rendered a higher corrosion resistance.

**Figure 13:**
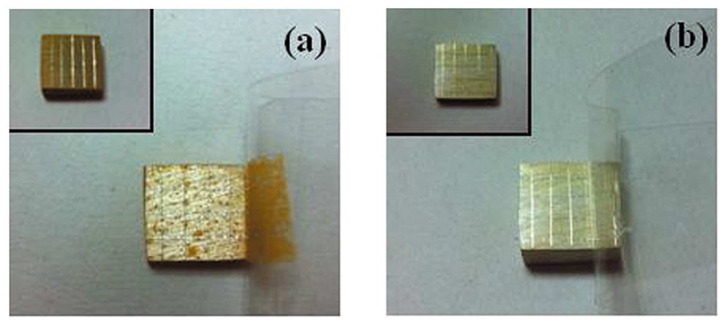
Optical photographs of (**a**) non-modified and (**b**) silane-modified cerium conversion coating before and after the cross cut tape test. Reprinted from [232] with permission from Wiley.

Along with the lack of self-healing ability, one of the main disadvantages of standalone RECCs is a rather poor adhesion of the conversion layer to Mg substrates. Kamde et al. [248] applied a CeCC on a Mg4Y alloy for different times of conversion treatment ranging from 30 to 1800 s. The increase in conversion time results in a significant deterioration of the adhesion between the RECC and substrate ranked from 4B to 1B (according to ASTM D3359-17 tape test). Figure 13a shows the result of a cross-cut test after depositing a cerium conversion layer from a CeCl_3_ solution containing H_2_O_2_ on an AZ31 alloy (dry adhesion). Clearly, a significant part of the conversion layer was removed by the 3M 600 tape [232]. Modification of the conversion bath with silane (bis-[triethoxysilylpropyl] tetrasulfide (BTESPT)) significantly improved the adhesion (Figure 13) [232]. Functionalizing the surface of RECCs with bridge molecules to enhance the adhesion of top coats has been achieved on substrates other than Mg such as steel [249]. The same approach is also expected to work for Mg substrates. Organic substances are added to RE conversion baths to improve the corrosion protection properties of the coating either by modifying the coating morphology or by its inhibition properties [220,221,250]. Although RECCs have a considerable positive effect on the corrosion protection of magnesium alloys, their limited adhesion, high price, and unsatisfactory long-term stability are the main issues limiting their commercialization. 

### 3.4. Vanadate-Based Conversion Coating

Vanadate conversion coatings form on the Mg surface as a result of vanadium oxide/hydroxide precipitation due to surface alkalinization when the magnesium alloy starts dissolving in the solution containing vanadium oxyanions. A higher alkalinization rate during the conversion coating step can lead to a higher vanadium oxide/hydroxide precipitation. Nabizadeh et al. [251] increased the alkalinization rate by adding Cu^2+^ into the conversion coating bath. Cu^2+^ ions can be reduced on the magnesium surface as metallic Cu, which possess cathodic characteristics compared to magnesium. Therefore, a higher alkalinization rate due to the cathodic reaction on Cu sites can be achieved. SST and polarization curves confirmed the improvement in the corrosion protection performance of the vanadate CC modified by Cu^2+^ ions [251]. 

Vanadate conversion coatings have been reported to be one of the most promising options for the replacement of chromate conversion coatings [123,252,253,254,255,256,257]. This is mainly due to the fact that vanadium-based conversion coatings have also shown self-healing properties

X-ray photoelectron spectroscopy (XPS) measurements from the surface of a vanadate conversion coating on magnesium alloys have detected the presence of both V(V) and V(IV) hydroxide/oxide [251,257]. Given that two different oxidation states of vanadium are available in the conversion coating, some studies have proposed a mechanism of protection similar to that of the chromate conversion coating [251,253]. 

In spite of the promising corrosion protection properties of vanadium-based conversion coatings, a recent study indicates that vanadates are almost as hazardous as chromates, being toxic if swallowed, suspected of damaging fertility, and toxic to aquatic life with long-lasting effects [258]. Given this, vanadate conversion coatings are not likely to find wide industrial application. 

### 3.5. Molybdate-Based Conversion Coating

**Molybdate** conversion coatings have been applied to steel, Zn, Al alloys, and tinplate. Only a few reports have described the application of molybdate conversion coatings to Mg alloys [228,229,259,260,261,262,263]. Unlike vanadates, as described above, molybdates are environmentally innocuous and may be considered as candidates for chromate replacement. Typically, the thickness of the molybdate films formed on Mg is lower than that on Al alloys and Zn. Furthermore, the coatings possess numerous micro-cracks. SiO_2_ nanoparticles [260], La (III) or F^−^ [229], Ce(III) [228] salts or phosphates [261] have been added to molybdate conversion baths to improve the coating morphology and protective properties. 

### 3.6. Stannate-Based Conversion Coating

**A stannate** conversion coating bath is usually alkaline (pH > 12) in contrast to other conversion coatings for magnesium alloys [86,191,264,265,266]. At such alkaline pH conditions, the formation of Mg(OH)_2_ considerably slows down the dissolution of Mg to take part in the formation of the conversion coating. Therefore, a relatively higher conversion bath temperature is used compared to that of the acidic conversion coating formulation in order to speed up the dissolution of Mg and the formation of a thicker conversion coating [86]. Magnesium tin oxide (MgSnO_3_.3H_2_O) is reported to be the main composition of the conversion coating. MgSnO_3_.3H_2_O precipitates mostly on the β phases of magnesium alloys in the form of hemispherical clusters [86,191]. 

The self-healing properties of stannate conversion coating have been reported in a few studies [267,268]. These studies mostly claim self-healing properties based on the SEM observation that shows filling of the formed pits on the magnesium alloys with corrosion products [267,268]. Although the stannate conversion coatings have been proven to significantly reduce the corrosion rate of magnesium alloys, a true validation of the self-healing properties of the coating requires more investigation. 

### 3.7. Selenite-Based Conversion Coating

Although the report of using a selenite-based bath for coating Mg can be traced back to a patent in the 1930s [269,270], only recently have studies re-opened the latent capabilities of selenite-based species on the modification of the Mg surface against corrosion [269,271,272].

Feng et al. [269] investigated Na_2_SeO_3_ as an inhibitor for AZ31 exposed to a NaCl solution. As a result of the interaction between the SeO_3_^2−^ ions and the Mg surface, a film was formed with a composition mostly made of MgSeO_3_ hydrate, Mg–Se oxyhydroxide, and selenium (Se^0^). The selenium metal appears in the film as the result of the reduction of selenite ions (SeO_3_^2−^) on the Mg substrate. The reduced selenium has been claimed to be in the form of an amorphous polymer network, which has been mechanistically compared to the structure of a chromium(VI) conversion coating [272,273] (see Section 3.1). A conversion bath with neutral pH (with selenite ions) [271] and an acidic bath (selenious acid) [272] also resulted in a conversion film of similar composition. 

As far as human health is concerned, the supernutritional level of selenium poses some risks, and industrial concentrations must be kept to a minimum [274,275,276]. However, since traces of selenium in the human body have been shown to be beneficial to several human physiological functions, a few researchers have recently studied selenium-based coatings on Mg for bioapplications [271,277]. In spite of the scant reports as to the protective performance of the selenite-conversion coating on magnesium, the nascent studies on its similarities to the CCC call for further investigation.

### 3.8. Magnesium Fluoride Conversion Coating

**Magnesium fluoride** conversion coatings are typically characterized by mediocre corrosion protection properties, but their adhesion is high. Due to the formation of a rather homogenous layer of MgF_2_ on the magnesium surface, it is also commonly used as one of the last pre-treatment steps for different types of subsequent coatings [278,279,280,281,282,283] (see in Table 3).

Passivation of magnesium by either alkaline fluoride or hydrofluoric acid occurs due to the formation of either partially hydrated magnesium fluoride MgF_2-x_OH_x_·yH_2_O or a mixture of Mg(OH)_2_ and MgF_2_ [99,284,285,286,287,288,289,290,291]. 

Due to the immediate passivation of the Mg surface, the conversion coating thickness remains between 0.1 and 2 µm. For instance, immersion of an AZ31 magnesium alloy in a 10% HF solution for one week led to the formation of a maximum 2 µm thick layer [92]. However, the conversion coating thickness could reach 10 to 200 µm if formed by substituting pre-existing Mg(OH)_2_ in concentrated HF. A higher MgF_2_/Mg(OH)_2_ ratio in coating composition typically results in higher corrosion protection ability. A more detailed recent review on fluoride conversion coatings on magnesium is available [292].

As mentioned in the pre-treatment section (0 in this review) in this review, HF can preferentially dissolve the Al-containing ß-phases in the magnesium alloy. Thus, microgalvanic corrosion between the ß- phases and magnesium matrix is also alleviated.

Although the fluoride-based conversion coatings are moderately protective, their assumed bio-compatibility has found wider application for bio- rather than engineering applications. The main disadvantage of this type of conversion coating is that its preparation implies the usage of concentrated hydrofluoric acid, which is highly toxic, fatal if swallowed, in contact with skin, or if inhaled [293]. 

### 3.9. Hexafluoro–Zirconate/Titanate/Hafnate/Niobate-Based Conversion Coating

**Figure 14:**
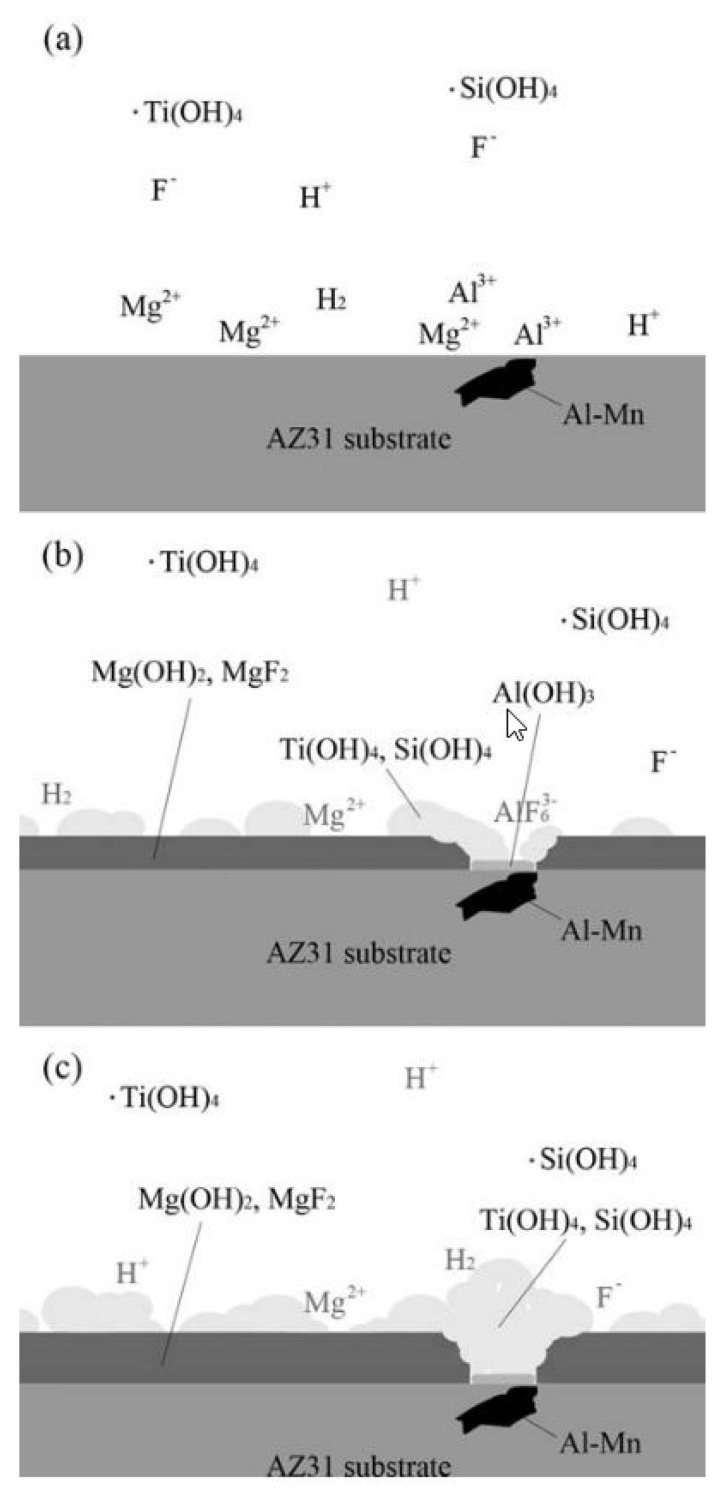
A schematic representation showing the titanate conversion coating formation on the AZ31 alloy: (**a**) the dissolution of Mg and Al as well as the discharge of hydrogen; (**b**) the formation of the porous layer composed of Mg(OH)_2_ and MgF_2_ as well as local precipitation of Si(OH)_4_ and Ti(OH)_4_ on top of the porous layer; and (**c**) the growth of the porous layer and the Si(OH)_4_ and Ti(OH)_4_ precipitates. Reprinted from [125] with permission from IOP.

**Hexafluoro–zirconate, –titanate, –hafnate,** or **–niobate** (H_2_ZrF_6_, H_2_TiF_6,_ H_2_HfF_6_, H_2_NbF_6_)-based conversion treatments were initially developed for aluminum alloys or metal-coated steels. A recent review of conversion coatings based on zirconium and titanium is available [294]. The understanding and development of thin film systems deposited from titanate, zirconate, hafnate, and niobate on magnesium substrates is still relatively limited [56,125,134,289,295,296,297,298,299,300,301,302,303,304,305,306,307,308]. The general formation mechanism of such conversion coatings implies the removal of a magnesium oxide/hydroxide film and the precipitation of amorphous oxides/hydroxides of the above-mentioned transition metals in response to the near-surface pH increase caused by the cathodic water reduction reaction on the Mg substrate. Thus, the film deposition is initiated on the second phases, on which the cathodic reactions occur [309]. The role of fluoride in the conversion bath is to maintain Zr, Ti, Hf, and Nb dissolved in the electrolyte until the pH is high enough for oxide precipitation. However, an excessive amount of fluorides may inhibit the conversion process due to the precipitation of MgF_2_. The resulting coatings that feature a thickness of several to ten nanometers on average may suffer from micro-cracks that become larger once the thickness of the conversion layer increases. Figure 14 and Table 2 demonstrate the main chemical reactions during the deposition of a titanate conversion layer on an AZ31 alloy with Al–Mn intermetallic.

Zr/Ti-based conversion coatings are usually referred to as *thin-film* coating technology. The formed film is usually 10–80 nm thick. The thickness of the coating depends on many parameters such as the microstructure and chemical composition of the substrate, conversion bath pH, and time of immersion [294]. For instance, the thickness of the oxide film formed in a Zr-based conversion solution on pure magnesium can exceed about ten times that of the film formed on pure zinc. 

Thin films deposited from the Ti/Zr-based conversion coatings provide minor stand-alone corrosion protection since the coatings hardly provide physical barrier properties. The formed film functions in combination with the subsequently applied (pigmented and non-pigmented) paint and offers remarkable corrosion inhibition and adhesion properties. They are compatible with additives such as (a) polymeric species for hybridization to obtain thicker films plus increased surface coverage; (b) adhesion-promoting molecules; (c) corrosion inhibitors; and (d) small additions of inorganic rare-earth/transition-metal cations for the refinery of the layer structure. Moreover, these films are compatible with Cr-based and phosphate-based systems to achieve all-in-one protection (by combination of advantages of these different based technologies).

The promoted adhesion between organic coatings and Ti/Zr-based conversion coatings on different metallic substrates has been demonstrated several times via macroscopic tests including the pull-off test and wettability test [310,311,312,313]. However, there are only a few fundamental studies on the chemical/physical bonding between the paint and the conversion coating on Mg and its alloys. Fockaert et al. [314] applied a model Zr-based conversion coating on thermally vaporized magnesium nano-layers, which resulted in a high (80–90%) surface concentration of hydroxides. After immersion of the metal surface in a 0.1 wt.% solution of dimethylsuccinate in tetrahydrofuran (THF), the chemisorption of dimethylsuccinate was investigated using attenuated total reflection-Fourier transform infrared spectroscopy (ATR-FTIR) in a Kretschmann geometry. The detection of carboxylate bonds on the Mg surface led to the speculation of a two-step chemisorption mechanism of dimethylsuccinate involving hydrolysis of the ester group forming carboxylic acid, followed by its deprotonation yielding the carboxylate anion. Interestingly, no carbonyl bonds were detected on the Mg surface, which suggests the full hydrolysis of dimethylsuccinate and its coordination into the Zr-based conversion coating. Both steps in the chemisorption mechanism can be activated by the presence of hydroxide end-groups. However, overhydroxylation can cause the deficiency of adsorption sites, which in turn leads to a decrease in the carboxylate bonds on the surface.

In another report by Fockaert et al. [315], a similar experimental setup was used to in situ investigate the interfacial bond formation of a commercial polyester-based primer and its degradation in deuterated water (D_2_O). The use of D_2_O as the corrosion medium allowed for better monitoring of the carboxylate stretching vibration in FTIR without being dominantly overlapped by the H_2_O signal. The results showed that the bonding mechanism of the primer on the Mg surface was similar for the native Mg surface and the treated Mg surface with the Zr-based conversion coating. However, the interfacial stability was improved after treatment with a Zr-based conversion solution, which resulted in 15 times higher delay time prior to disbondment of the carboxylate groups.

The conversion coatings based on Ti/Zr are highly sensitive to substrate conditions and require accurate process operation. It stems from the fact that substrate etching during the coating process is low. The coating process cannot compensate for inadequate cleaning, degreasing, or other surface pre-conditioning. If the content of alloying elements results in a high density of intermetallic particles, their removal from the surface via a de-oxidizing step is particularly important.

### 3.10. Organic-Based Conversion Coating

**Figure 15:**
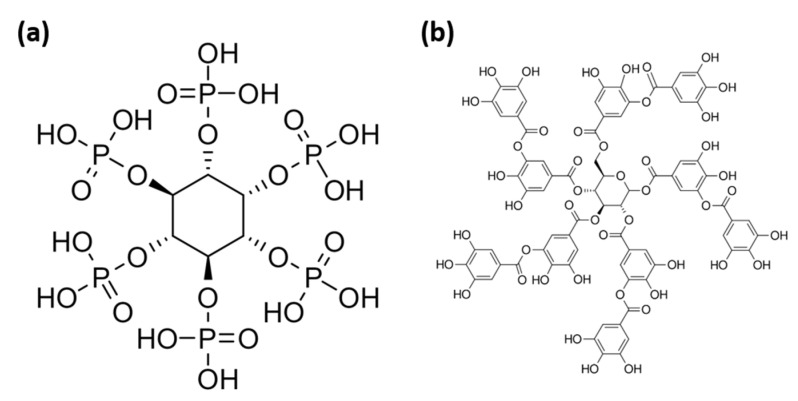
Molecular structure of (**a**) phytic acid and (**b**) tannic acid.

The application of **phytic acid**, also known as *D*-myo-inositol-1,2,3,4,5,6-hexakisdihydrophosphoric acid, for conversion coatings on several metals including magnesium has been gaining ground for the last several years. The “myo” prefix indicates a particular orientation of the hydroxyl groups with respect to the inositol ring, while the phosphate groups are not bound to each other. This acid is an ester of a hexabasic alcohol myo-inositol and six phosphoric acid residues (Figure 15a) [316]. The application of phytic acid as a corrosion inhibitor for several metals including Cu, Mg, Zn, Al, and steel has been recently summarized by Kuznetsov [316]. One of the first applications of phytic acid to form a conversion layer on Mg was reported by Liu et al. [317]. The reported superior corrosion protection properties compared to a PCC and a CCC have drawn considerable attention, resulting in numerous studies to find more organic-based conversion coating alternatives to hazardous CCCs.

The general mechanism of the formation of a conversion coating based on phytic acid relies on the formation of insoluble chelating compounds with Mg^2+^ ions, released in the acidic condition of the conversion solution. One Mg^2+^ ion can bind to more than one phytic acid, which may lead to the formation of a cross-linked structure of phytic acid with Mg^2+^ connectors [318]. Other alloying elements such as Al in AZ91 are also present in this network [319]. The free hydroxyl groups of the phytic acid molecules are also believed to facilitate the adhesion to the subsequent organic coating, as an excellent adhesion has been reported for a commercial epoxy resin [238,318,320,321].

**Figure 16:**
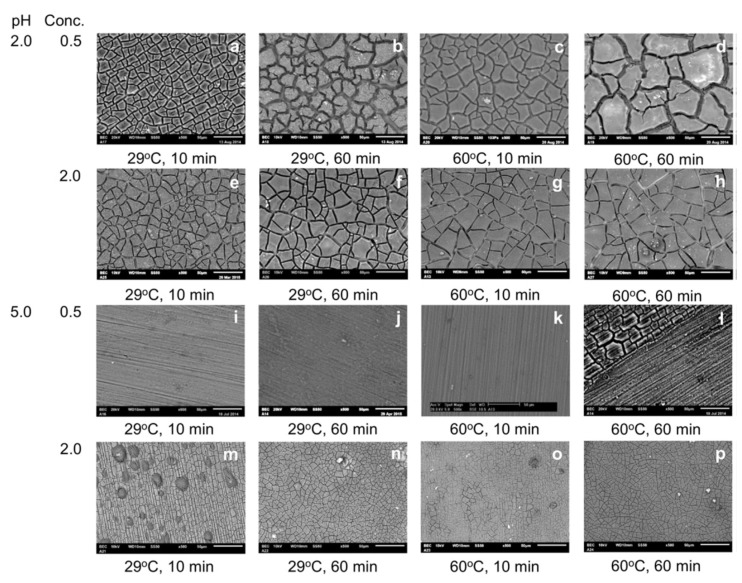
SEM images of the phytate conversion coatings on the AZ31 alloy samples treated in phytic acid solutions, varying pH, phytic acid concentration, treatment time, and temperature. Reprinted from [324] with permission from Elsevier.

The processing parameters for phytic acid conversion coating including pH and concentration of phytic acid, treatment time, and temperature to obtain the best performing protective coating have been the subject of several reports [319,322,323,324]. An overview of phytate conversion coatings applied to Mg alloys, together with the statistical design of corresponding coatings on the AZ31 alloy has recently been provided by Hernandez-Alvarado and co-workers [324]. The optimal parameters to obtain a corrosion protective phytic acid conversion layer on the AZ31 alloy have been reported to be 0.5%.v/v, pH = 5, treatment times of 10–30 min, and a treatment temperature of 29 °C [324]. The conversion bath pH plays a significant role on the thickness and morphology of the phytate conversion layer. On one hand, the electrolyte pH affects the Mg stability and the concentration of Mg^2+^, and on the other hand, it determines the number of deprotonated hydroxyl groups on a phytic acid molecule that can readily bind to Mg^2+^ ions. A thicker conversion coating featuring more mud-like cracks can be seen in Figure 16 for the corresponding pH of 2 compared to that of pH 5. More aggressive H_2_ evolution at more acidic conditions is believed to be one of the main factors for the crack formation in the deposited layer. As a result, a lower corrosion protective performance has been recorded for the conversion coatings generated in a relatively low pH electrolyte. On the other hand, at alkaline conditions, a very low production of Mg^2+^ leads to a very low deposition of the phytate conversion layer, which in turn, results in inferior corrosion protection compared to the acidic condition [325]. Moreover, at pH higher than 9.0, the conformation of phytic acid changes [326]. Therefore, the Mg^2+^-phytate bonds formed at alkaline conditions may break after exposure to neutral conditions, which means the partial disintegration of the Mg-phytate network [325].

**Tannic acid (TAc)**, a type of polyphenol with the ability to form complexes with different cations via its hydroxyl groups (see Figure 15b), has been reported to yield a conversion coating on an AZ91D alloy with high protective properties [304,305,306]. Chen at al. used a tannic acid base conversion solution containing NH_4_VO_3_, K_2_ZrF_6_, and H_3_PO_4_ to apply a conversion coating on the AZ91 alloy [304,305]. A better performance in SST compared to a CCC has been reported. It was suggested that the tannic acid is first hydrolyzed to gallic acid and then oxidized to penta-hydroxy benzoic acid, which forms a complex with Mg^2+^. As a result, an organic polymer with a mesh structure and filled with MgF_2_ and Al_2_O_3_ is formed. The V^5+^ ions in the conversion solution participate in the oxidation of gallic acid. In spite of the promising performance, the conversion bath contains considerable amounts of toxic NH_4_VO_3_ and Na_2_B_4_O_7_ (the latter has been included by the European Chemical Agency, ECHA, in the candidate list of substances of very high concern for authorization [327]). Applied directly to several grades of pure Mg and industrially relevant alloys, tannic acid only showed a positive effect in the case of low-quality Mg with active Fe contamination [111]. 

A conversion coating formed on a magnesium alloy using a single component **TAc** (as well as its building block, gallic acid) has seldom been reported. This is probably due to an uncontrollable deposition and highly defective precipitated layer. Wang et al. [283] applied a MgF_2_ thin layer prior to the conversion coating with **TAc** to control the substrate dissolution rate and homogenize the magnesium substrate electrochemical activity. Out of different **TAc** concentrations (ranging 0.5–5 mg·mL^−1^) and conversion bath pH values (ranged 5.5–10), the **TAc** coating that formed at a pH of 10 and concentration of 2 mg.mL^−1^ resulted in the most uniform and dense morphology on the surface.

**Figure 17:**
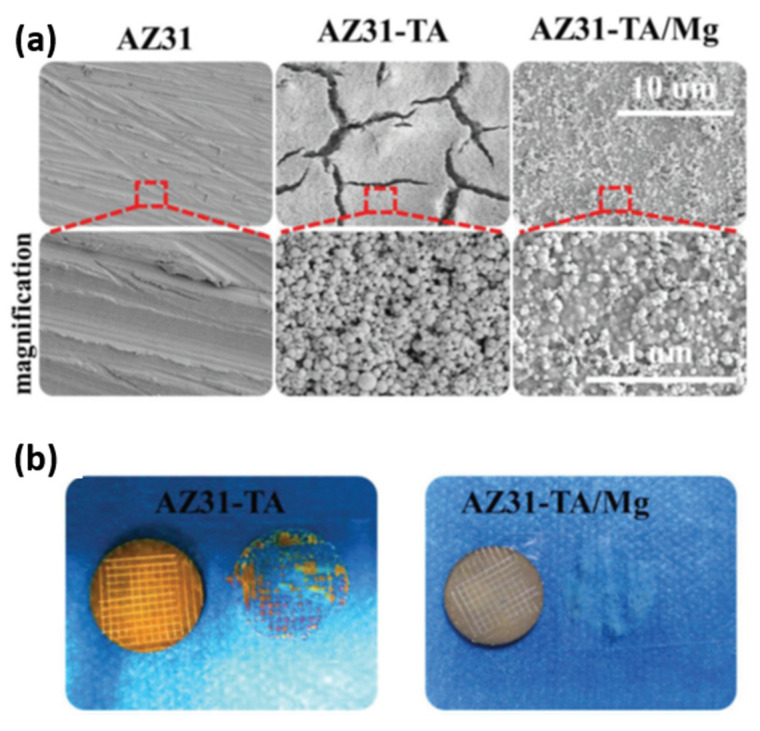
Surface morphology of AZ31, AZ31-TA, and AZ31-TA/Mg. (**a**) SEM morphologies, (**b**) tape test on AZ31-TA and AZ31-TA/Mg using Scotch tape. Adapted and reprinted from [328] with permission from John Wiley & Sons.

In another approach to overcome the highly defected morphology of the **TAc** conversion coating, Zhang et al. [328] prepared a layer-by-layer (LBL) assembly of magnesium-**TAc** complex layers on an AZ31 alloy. In addition to the Mg^2+^ supplied from the dissolution of the Mg substrate, Mg^2+^ was supplemented in the conversion solution via the addition of MgSO_4_. Thus, the extra Mg^2+^ ions act as crosslinking points, leading to higher connection points between the applied **TAc** layers. Figure 17a shows the considerably denser and less cracked morphology of the 5-layer LBL assembly on AZ31 obtained from a Mg^2+^-containing **TAc** conversion solution (marked AZ31-TA/Mg in the figure) compared to that without Mg^2+^ (marked AZ31-TA in the figure). Furthermore, the stronger bonds between the layers and the better integrity of the coating are reflected in the adhesion test results (based on ASTM 3359-02) presented in Figure 17b. The same approach of LBL assembly with Mg^2+^-rich organic conversion solution was conducted for another chelating molecule, epigallocatechin gallate (EGCG), which is the most abundant catechin in tea. Apart from the reported corrosion protection, the green chemistry of such a coating makes it suitable for bioapplications [329]. 

There are also a few more recent reports on the use of other small organic molecules as the precursor for the formation of a conversion coating on magnesium. Abatti et al. [330] used vanillic acid (4-hydroxy-3-methoxybenzoic acid) solution to form a thin layer (~60 nm) of mixed magnesium vanillate and MgO/Mg(OH)_2_ on an AZ31 magnesium alloy. The surface of the AZ31 was pre-treated with NaOH to provide a higher concentration of Mg(OH)_2_ on the surface, which was expected to result in a higher rate of interaction between vanillic acid and Mg^2+^ ions. The conversion coating resulted in two orders of magnitude reduction in the corrosion current density and strong passivation behavior according to the dynamic polarization test. In addition to the stand-alone corrosion protection of the conversion coating, the adhesion of a polymer top coat (poly(4-vinylpyridine)) was significantly enhanced, which was attributed to the hydrogen bond and/or dipole interaction due to the presence of hydroxyl and methoxy groups of vanillic acid. 

A post-immersion of coated metal in a solution containing surfactants is a common approach to achieve hydrophobicity, which eventually enhances the corrosion protection performance of the coating (see Section 3.12). However, the use of surfactants as the main precursor of a conversion coating has barely been reported. Frignani et al. [331] applied a series of conversion coatings containing sodium salts of mono-carboxylic acids with alkyl chain lengths between 12 and 18. The formed conversion coating process required a fairly long time (more than 24 h of immersion), which could be reduced at an elevated bath temperature. The highest corrosion protection performance was achieved with the highest alkyl chain length, which was speculated to be due to the higher insolubility and hydrophobicity of the formed coating.

**Ionic liquids (IL)** are salts with low melting points (below 100 °C) [332], which are mostly composed of organic cation–anion pairs [333,334]. They feature unique physiochemical properties such as high conductivity (0.1–14 mS/cm), high thermal stability, broad electrochemical stability window, and low volatility [335]. The organic nature of the cation–anion pair leads to boundless formulations of the ILs, which can be “designed”’ according to the desired properties. However, only a very limited number of IL formulations have been studied for conversion coating electrolytes on Mg, probably due to the relatively high cost and the necessity of special care of the atmosphere for handling. 

The trihexyl(tetradecyl)phosphonium cation ([P_6,6,6,14_]) is one of the widely used cation components of the ILs, specifically studied to form a conversion coating on Mg alloys. It possesses high thermal and electrochemical stability [336] with a wide stability potential window of −3.2 V (vs. Fc^+^/Fc), which makes it electrochemically inactive in contact with the Mg surface. Other studied cations in ILs for conversion coatings include trioctylammonium [N_888_H] [337], imidazolium-based [338,339], and pyrrolidinium-based [338]. 

The anion part of the ionic liquids is the main precursor participating in the formation of the conversion layer. Among the variety of anions of ionic liquids used for the conversion coating on Mg, bis(trifluoromethanesulfonyl)imide (NTf_2_, also abbreviated as TFSA in the literature) is one of the most investigated anion due to its promising anti-corrosion results [340]. However, the relatively high cost of NTf_2_ and the environmental impact of the potentially toxic fluorine in NTf_2_ [341] have led researchers to seek alternatives such as phosphinate/phosphate-based anions and their sulfur analogs [332,338,340,342,343,344,345,346]

The conversion layer on Mg formed in IL is generally characterized as a thin film (below micron size) compared to most of the typical aqueous conversion coating. This is principally due to the low reactivity of the Mg in a non-aqueous and electrochemically stable media like IL. 

The addition of water to an IL can electrochemically activate the Mg surface and promote the formation of a conversion coating [342]. Nevertheless, the presence of water in IL can adversely affect the corrosion protection properties of the formed film at prolonged treatment times due to the excessive reaction of the Mg substrate with water [340]. Notably, the reproducibility of the experiment strongly relies on the careful handling of the exposure of the IL to the atmosphere and humidity.

The film formation involves the physisorption/chemisorption of the IL anions on Mg(OH)_2_/MgO/Mg present on the Mg substrate as well as their chemical/electrochemical interaction with the Mg substrate. Hypothetically, the physisorption of the anions requires charge neutrality, which is met by the electrostatic interaction with the present cations.

In the case of fluoride-containing anions such as NTf_2_, the fluoride salt of magnesium and other alloying elements are also present in the formed film [347,348]. The fluoride F^−^ ion is produced from the NTf_2_ decomposition into smaller molecule fractions [337,349]. The decomposition rate is accelerated with the increase in temperature. Therefore, applying an elevated temperature (up to 300 °C has been reported [349]) during the exposure of the Mg surface to the IL is a common way to enhance the film formation rate. For the phosphate-based anions, the bond between Mg and the phosphate functional groups has also been deduced from XPS [349].

Diluting the ionic liquids with organic solvents and the anodic polarization of the Mg substrate during the conversion coating process are two more approaches to enhance the kinetics of the conversion coating formation [344,348,350].

Deep eutectic solvents (DES) are a type of ionic liquid with the advantages of having lower toxicity, lower cost, and a more straightforward preparation process, which make them more promising in industrial applications [351]. In a recent study by Guo et al. [352], a relatively thick conversion coating (~2 µm after 60 min of immersion) was formed on an AZ31B alloy in a DES, based on choline chloride–urea. Clear evidence of MgH_2_ was found in the formed conversion coating [353]. The mechanism of the film formation was hypothesized as the reaction of Mg with the decomposition product of urea, which leads to the formation of MgH_2_, MgO, and MgCO_3_. Although the formed conversion coating did exhibit minimal corrosion protection properties due to its porous structure, it could trap more epoxy coating during the dip-coating process and improve the overall corrosion protection properties of the epoxy-coated substrate. 

A literature review of the limited reports on the performance of stand-alone ILs and DESs conversion coatings against corrosion yielded a generally unsatisfactory outcome. The corrosion tests were mostly reported either against a mild corrosive medium such as diluted NaCl solutions or just after a short exposure time. 

### 3.11. Layered Double Hydroxide (LDH)

**Layered double hydroxide (LDH)** including hydrotalcite and hydrotalcite-like compounds is a layered structure containing anions that are loosely intercalated between hydroxide layers of metal cations [354]. LDH obey the general formula: [M_1−m_^2+^M_m_^3+^ (OH)_2_]^m+^ [(A^n−^)_m/n_·xH_2_O]^m−^, with M^2+^ being a divalent cation (e.g. Mg^2+^, Zn^2+^, etc.), M^3+^ being a trivalent cation (e.g. Al^3+^, Fe^3+^, etc.), and *m < 1* (typically 0.2 or 0.33) is the degree of substitution of M(OH)_2_. Given the partial substitutions of M^3+^ for M^2+^, the hydroxide layers are positively charged and are intercalated with anions (A*^n^*^−^, e.g. CO_3_^2−^, NO_3_^−^, Cl^−^, SO_4_^2−^, PO_4_^3−^, VO_3_^−^, etc.) to maintain the overall charge neutrality. M^+^M^3+^ LDH is also known as Li–Al LDH [355,356]. LDH particles loaded with corrosion inhibitors as charge compensating anions have been widely investigated as potential active anticorrosion pigments for different paint formulations. LDHs are inherently of high interest for corrosion inhibition due to their ability to release inhibitors in response to corrosion-related stimuli. Anion-exchange characteristics of LDH provide a second positive functionality: the trapping of corrosive species [357,358]. Even without any incorporated inhibitor anions, LDHs are reported to improve the corrosion resistance by entrapping corrosive anions such as Cl^−^ [359]. Cerium cations can also be incorporated in the hydroxide layer [360,361,362] and big organic molecules such as 5,10,15,20-tetra(r-sulfonatephenyl)-prophyrin can be asymmetrically intercalated with their hydrophilic functional groups [363]. 

**Figure 18:**
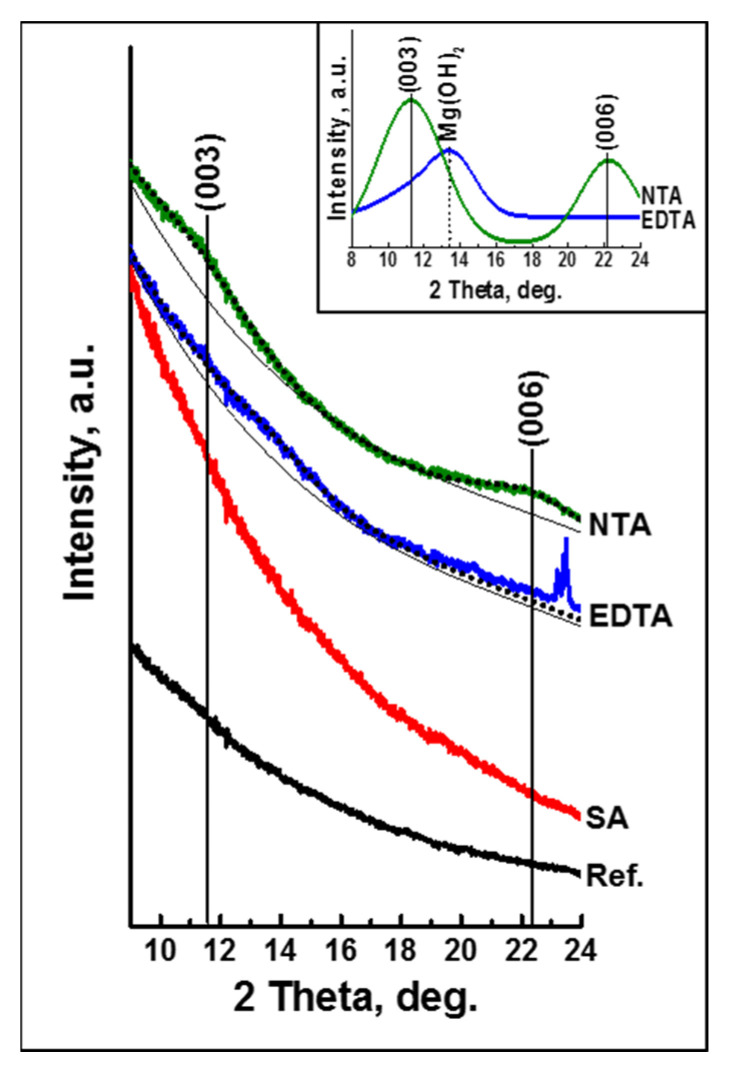
Grazing incidence XRD pattern of the AZ91 surface after 48 h in the reference, salicylic acid, EDTA, and NTA sodium salt solutions at room temperature. The data were shifted vertically for clarity. The inset shows the Gaussian polynomial fit of the NTA and EDTA patterns. Reprinted from [385] with permission from Springer Nature.

Applying LDH directly on engineering metals such as aluminum and steel as a conversion layer has been studied extensively over the past decades [355,357,358,360,364,365,366,367], but the application of LDH for protecting Mg alloys has only recently attracted the attention of the scientific community. Several research reports have been undertaken to investigate the inhibitory function of LDH coatings on magnesium alloys. A review of the synthetic routes of LDH growth on Mg alloys is available in [368]. Typically, LDH is grown at high temperatures and pressure conditions (95 °C and in autoclave). A number of recent studies have used LDH to seal a porous layer formed by the PEO coating method [369,370,371,372,373]. Other studies have focused on the precipitation of Mg–Al or Zn–Al LDH from the electrolyte and aimed to modify the process parameters and fabrication approaches to obtain more adherence to the substrate layer with better morphology [369,374,375,376,377,378,379,380,381,382,383]. Mg–Al LDH loaded with either phytate [379], molybdate [384], or vanadate [369] have been reported. Typically, LDH is formed from the Al^3+^/Mg^2+^ containing electrolyte and precipitates on the Mg surface at high pressure, usually in an autoclave. Recently, a step forward has been made by succeeding in intrinsic in situ growth of Mg–Al LDH at ambient pressure [385,386]. The AZ91 alloy was introduced into Al^3+^ containing aqueous electrolyte containing organic additives (e.g. sodium salt of nitrilotriacetic acid (NTA) [385]) and then the more environmentally benign diethylenetriaminepentaacetic acid (DTPA) pentasodium salt [386]. Superficial dissolution of the alloy generated Mg^2+^ cations, followed by their precipitation as Mg–Al LDH, which greatly improves the adhesion of grown layers and hence their protective ability. Moreover, it has been shown that in the presence of the sodium salt of nitrilotriacetic acid, Mg–Al LDH even grew at room temperature [385] (see Figure 18).

The direct growth of Mg–Al LDH on the PEO-treated AZ91 alloy at atmospheric pressure was demonstrated in [373]. This was facilitated by organic additives, namely a combination of the strong complexing agent DTPA (diethylenetriamine-pentaacetate sodium) binding Mg^2+^ and Al^3+^, and salicylate, which competitively adsorbs on the PEO surface and counterbalances the dissolution of PEO. Following the steady growth of the LDH layer, the pores of the PEO layer were sealed. 

The LDH resembling material, dawsonite NaAlCO_3_(OH)_2_, has been reported to possess high corrosion protection abilities applied on the AZ31 alloy [387]. 

### 3.12. Post-Treatment on Conversion Coatings

Post-treatment methods are adopted to improve the corrosion resistance of conversion coatings or to functionalize the surface for a specific application such as aesthetics or bioapplications. Hydrothermal post-treatment was carried out aiming to modify the coating morphology and chemistry. The influence of hydrothermal post-treatment on conversion coatings is specific to each conversion coating and the post-treatment method. Examples of hydrothermal post-treatment have been provided in the corresponding conversion coating section.

Endowing a hydrophobic characteristic to a conversion coating via exposure to a surfactant-containing solution is also a common approach to achieve higher corrosion resistance. The post-treatment procedure involves a simple immersion of the coated sample in a surfactant-containing solution. The most studied surfactant is stearic acid (STA), which has been applied on various conversion coatings including Mg(OH)_2_ formed by hydrothermal treatment [388,389,390], LDH [391,392], CaP conversion coating [199,393,394], phytate conversion coating [395], stannate conversion coating [396], Cr(III) conversion coating [135], and CaF_2_/MgF_2_ conversion coating [280]. 

The hydrophobicity provided by stearic acid leads to a significant increase in corrosion resistance by repelling the aqueous corrosive medium away from the sample surface. High surface roughness and porous morphology of conversion coatings such as LDH further enhance the water repellent characteristics of the STA modified surface, since more air-pockets are trapped within the coating [199,392]. In spite of the numerous available surfactants, only a few other than STA have been studied to endow conversion coatings with hydrophobicity [397]. Thus, considering a remarkable improvement in the corrosion resistance of conversion coatings via a simple immersion method, hydrophobization via a post-treatment deserves more attention and further investigation.

An overview of the selected conversion formulations and coatings is presented in Table 3. The details of the conversion coating processes and the testing media are provided. It is worth mentioning that the evaluation of the corrosion protection performance of a conversion coating must not be solely based on comparing the corrosion test results such as the corrosion current, weight loss, impedance value, corrosion potential, etc. In order to conduct a fair comparison, it is important to take into account all of the factors involved including the testing conditions, the method of evaluation, and the substrate. The performance of a conversion coating can be altered when combined with different primers. Corresponding compatibility for a conversion coating with a primer or a topcoat should be verified case by case. As a well-established fact, the concentration of Cl- ions in the testing solution can significantly affect the corrosion rate of magnesium alloys [398,399]. Moreover, for a conversion coating process to be considered for the replacement of a CCC, it is vital to consider the cost of the conversion bath, the stability of the bath, the complexity of the process as well as the post-treatment of the conversion chemical wastes.

**Table 3:** Overview of the selected conversion coating procedures reported in the literature.

Treatment	Pre-Treatment	Bath Component/ Concentration/ Mechanism	Initial Bulk pH	Duration, Bath T	Thickness, Surface Composition/ Morphology	Performance	Testing Medium	Alloy	Reference/ Year	Advantage/ Disadvantage
**Cr(VI)**	Alkaline decreasing Nitric acid pickling Chromic acid pickling HF activation	K_2_Cr_2_O_7_ 40 g/L K_2_SO_4_ 20 g/L	n/a	1–14 min 75 °C with air bubbling	11 µm Cr_2_O_3_, Cr(OH)_3_, K_2_CrO_4_, MgO	E_corr_ shift −1.61 to –1.3 V i_corr_ decreased from 0.079 to 0.02 A/m^2^ for bare to CCC coated substrate	1% NaCl	EV31A	[80] 2014	Highly effective, with self-healing effect, abrasion resistance, commercially available, one step process, long bath life / carcinogenic, toxic, banned by EU regulations, urgent need for replacement
**Cr(III)**	Grinding to 1000, ultrasonication in acetone	(0.3 M CrCl_3_ 0.05 M NH_4_H_2_PO_2_) in choline chloride:ethylene glycol (1:2)	n/a	30–60 min 30 ± 5 °C under ultrasonic treatment followed by methanol rinsing	3 µm Cr_2_O_3_ Microcracks	E_corr_ shift −1.51 to –1.45 V i_corr_ decreased from 609 µA/cm^2^ to 1.25 µA/cm^2^ for bare to Cr(III)CC coated substrate	3.5% NaCl	AZ31	[135] 2016	Commercially available, robust and easy bath maintenance and process control, one step process / moderate corrosion protection, weak self-healing properties, contains minor amount of carcinogenic Cr(VI)
	Pickling/activation processes according to SAE-AMS-M-3171	e.g., 1–5 g/L Cr_2_(SO_4_)_3_, 1–5 g/L K_2_ZrF_6_, 0–5 g/L MeBF_4_ 0–5 g/L ZnSO_4_ 0.5–1.5 g/L soluble cellulose 0–10 g/L surfactant	3.7–4.0	5–15 min Ambient temp up to 50 °C	Adhesion 2–2.5 times higher than for DOW 7 chromate treated process	n/a	n/a	AZ91C-T6 ZE41-T5	[136] 2010	
**Phosphate**	Alkali washing in 60 wt.% NaOH, grinding to 1200 grit, cleaning in pure water and ethanol	35 g/L Mn(H_2_PO_4_)_2_, 0.5 g/L of NaF or C_6_H_5_Na_3_O_7_ or C_6_H_8_O_7_	2.5	1 s–20 min 95 °C	Lamellar structure with block particles. Intermediate layer: Mg_3_(PO_4_)_2_, AlPO_4_, and Mg(OH)_2_. Outer layer: MnHPO_4_.	E_corr_ shift from −1.5 to −0.34, −0.468, and −1.37 V for the bare to phosphated substrate containing citric acid, NaF and Na-citrate, respectively. I_corr_ reduction from 460 µA/cm^2^ to 5 nA/cm^2^_,_ 32 nA/cm^2^ and 5 µA/cm^2^ for the bare to phosphated substrate containing citric acid, NaF and Na-citrate, respectively.	3.5 wt.% NaCl	AZ31	[205] (2013)	Commercially available, eco-benign, good adhesion with paint, / moderate corrosion protection effect, requires elevated temperatures, multi-step process, requires accurate operation, bath maintenance and control due to low stability of bath’s pH
	Grinding to 1200 grits, cleaned with industrial alcohol in ultrasonic bath, degreased in NaOH, acid pickling in mixture of HF and C_2_H_6_O_2_	4–36 mL/L H_3_PO_4_, 40–90 g/L Ba(H_2_PO_4_)_2_ 1–3 g/L NaF	n/a	10–30 min, 60–100 °C	Mg, MgO, and some amorphous phases	Corrosion spots appear after 20 h of SST for the phosphate conversion coated sample, while white massive corrosion blocks after 8 h of SST covered the untreated sample	SST, damp heat test	AZ31	[400] (2009)	
	Grinding to 4000 grits in ethanol, rinsed in ethanol	0.1 M Mg(OH)_2_, 0.24 M H_3_PO_4_	3.2	20 min, 45 °C	2.5 µm thick coating after 20 min of phosphating. Micro-cracks structure. Coating composed of MgO/Mg(OH)_2_ and Mg-PO_4_ compounds	E_corr_ shift from −1.61 V to −1.41 V for the bare to phosphated substrate. I_corr_ reduced from 223 µA/cm^2^ to 6.9 µA/cm^2^ for the bare to treated substrate. Pit initiation time was delayed from 10 min to 24 h for the bare to the phosphated substrate.	0.1 M and 0.05 M NaCl	AZ31	[401] (2017)	
	Grinding to 2000 grits in ethanol, rinsed in ethanol	Step 1: H_3_PO_4,_ step 2: 0.05 M (NH_4_)_2_HPO_4_	Step1: 5 Step2: n/a	Step 1: 30 min, 40 °C Step 2: 30–60 min, 80 °C	Inner layer: MgHPO_4_.3H_2_O Outer layer: MgNH_4_PO_4_.6H_2_O	Corrosion resistance of phosphated substrates is about 20 times better than untreated samples. E_corr_ shift from −1.6 V to −1.53 V for the bare to 2-step treated substrate. I_corr_ reduction from 63 µA/cm^2^ to 3.7 µA/cm^2^for the bare to 2-step treated substrate.	SBF	AZ31	[402] (2015)	
	grinding to 2500 grits, degreased in absolute ethanol, acid pickling in HNO_3_ and then HF solution. Rinsed by distilled water between each step	10 g/L Y(NO_3_)_3_ then in NH_4_H_2_PO_4_ bath with concentration of 1–2.5%	n/a	30–180 s, 75–90 °C	Y_2_O_3_, YO_x/y_, Mg_3_(PO_4_)_2_, AlPO_4_ and YPO_4_	E_corr_ shift positively about 180mV compared to the uncoated one at, I_corr_ reduced from 70.2 µA/cm^2^ to 7.7 µA/cm^2^ for bare to conversion coated sample	3.5% NaCl	AZ91	[403] (2016)	
**Phosphate-permanganate**	Grinding to 1500 grit, polishing with 0.3 µm Al_2_O_3_ paste, pure water cleaning, alkaline degreasing with NaOH + Na_3_PO_4_, pure water cleaning, acid pickling with H_3_PO_4_, surface activation with HF	20 g/L KMnO_4_, 60 g/L MnHPO_4_	n/a	10 min, 50 °C	Network-like cracks in coating containing metal oxides (Mg, Mn and Al), Hydroxide, phosphates and spinel for AZ series alloy	Equivalent or slightly better passive capability than the conventional Cr^6+^-based conversion treatment of AZ series alloys, but an inferior capability for the pure Mg	5 wt.% NaCl	AZ61,AZ80, AZ91, and pure Mg	[187] (2002)	
	Grinding to 2000 grit, rinsed with DI water, cleaned in acetone, dried in a stream of hot air	0.87 M NH_4_H_2_PO_4_, 0.063–0.51 M KMnO_4_	n/a	10 min, 60 °C	Three layer: 1- porous layer on substrate 2- compact intermediate layer 3: cellular overlay. Thickness in the range of 8–1 µm	Less than 10% corroded fraction after 24 h SST for the phosphate solution containing 0.51 M KMnO_4_, while more than 50% of bare AZ31 was corroded after 24 h	Solution of 0.05 M NaCl and 0.10 M Na_2_SO_4_. SST (ASTM B117)	AZ31	[189] (2013)	
	Degreasing with ethanol, acid pickling with H_3_PO_4_, tap water rinsing, NaOH activating, tap water rinsing	100 g/L NH_4_H_2_PO_4,_ 30 g/L KMnO_4_	3.5	40 °C	First layer: homogenous but with many cracks/ Second layer: nodules of Mn-rich oxides	Reduction in corrosion rate from SST by phosphate-permanganate conversion coating. +200 mV shift to E_corr_ and two orders of magnitude reduction in i_corr,_ comparing untreated and phosphate-permanganate coated sample	Salt spray ASTM B117, Electrochemical tests in solution containing Na_2_SO_4_, NaHCO_3_ and NaCl (pH 8.2)	AZ91 and AM50	[404] (2010)	
	Grinding to 1200 grits, DI water, air stream drying	0.1 M KMnO_4_ 0.025 M Mn(NO_3_)_2_ 0.02 M KH_2_PO_4_	1.7	90 s 25 °C	230 nm nearly crack-free	E_corr_ shift –1.56 V to –1.41 V i_corr_ decreased from 20 µA/cm^2^ to 1.6 µA/cm^2^ for bare to PCC coated substrate compared to 0.4 µA/cm^2^ for DOW1 CCC Sufficient electrical conductivity, Poor crystallinity	0.05 M NaCl + 0.1 M Na_2_SO_4_	AZ31	[81] (2015)	
	Blasting (alumina F220-500), degreasing (e.g. NaOH), pickling (H_3_PO_4_), activating (or HF)	0.2 M KMnO_4_ 0.1 M Na_3_PO_4_ 2 g/L Ca(NO_3_)_2_ 2 g/L Y(NO_3_)_3_	2.5–5 H_3_PO_4_	n/a	n/a	CC treated uncoated samples withstood 168 h of SSF and 500 h of humidity test, CC treated samples coated with primer and resin withstood 2000 h of SSF, Good adhesion of organic coats	SSF (ASTM B117 Sec. 8.1 and 10.1); Humidity tests; Cross-cut adhesion tests	EV31A, AZ91, AM60	[405] 2015, 2017	
**Zinc** **-phosphate**	Grinding to 1000 grits, degreased in absolute acetone, rinsed by DI water	Primary bath: 2 g/L ZnO, 12 g/L H_3_PO_4_, 1g/L NaF, 4 g/L C_4_H_4_O_6_Na_2_, 6 g/L NaNO_3_, 0.5 g/L Na_4_P_2_O_7_ + 2 g/L nano-CeO_2_ or 2 g/L nano-ZnO or 2 g/L nano-ZrO_2_	n/a	60 min, 60 °C	n/a	I_corr_ reduction from 1.24 mA/cm^2^ to 0.06 mA/cm^2^ for original phosphate coated to the nano-CeO_2_ modified coating. E_corr_ shifted from −1.42 V to −1.30 V for original phosphate coated to the nano-CeO_2_ modified coating. Significant reduction in crack ratio and size on the nano-CeO_2_ modified coating compared to the original phosphate coating	3.5% NaCl	AZ91D	[406] (2017)	
	Grinding to 2000 grit, degreased in KOH, rinsed in distilled water	1 M H_3_PO_4_, 0.004–0.068 M Zn(NO_3_)_2_.6H_2_O, 0.042 M NaNO_2,_ 0.021 M NaNO_3,_ 0.024 M NaF, 0.034 M Na_2_HPO_4_.12H_2_O	2.1–4	50 °C	Outer porous hopeite crystal and inner dense amorphous compound	I_corr_ reduction to 50 time lower value from bare to the treated sample at pH of 3.07	0.5 M NaCl	AZ31	[175] (2013)	
	Grinding to 3000 grits, alkaline degreasing, acid pickling	50 g/L Zn(H_2_PO_4_)_2_, 20 g/L NaH_2_PO_4_, 30 g/L 50% Mn(NO_3_)_2_, 5 g/L C_6_H_8_O_7_, 0.2 g/L C_18_H_29_NaO_3_S	1.8–2.6	15 min, 45 °C	Homogeneous and ordered crystals containing Zn_3_(PO_4_)_2_ and MnHPO_4_. Some cracks	E_corr_ shift from −1.571 V to −0.370 V for bare to coated substrate in phosphate solution of pH 2. I_corr_ reduction from 129 µA/cm^2^ to 5 µA/cm^2^ for bare to coated substrate in phosphate solution of pH 2.	3.5% NaCl	Mg-8.5Li	[204] (2014)	
	Heat treatment of samples for 0–24 h at 400 °C, grinding to 2000 grits, cleaned with distilled water, degreased in KOH, rinsed in distilled water	12.4 g/L H_3_PO_4_ (85 wt.%), 5 g/L Zn(NO_3_)_2_.6.H_2_O, 20 g/L NaH_2_PO_4_.12H_2_O, 3 g/L NaNO_2_, 1.84 g/L NaNO_3_, 1 g/L NaF	3–3.2	50 °C	Inner layer of MgZn_2_(PO_4_)_2_ and Mg_3_(PO_4_)_2_. Outer layer of hopeite (Zn_3_(PO_4_)_2_·4H2O)	the sample with 24 h heat treatment withstood 24 h in immersion test, While the bare sample withstood only 3 h in immersion test	0.5 M NaCl	AZ91	[407] (2013)	
	Grinding to 1000 grits, rinsed with DI water, degrease in alcohol	2 g/L ZnO, 12 g/L H_3_PO_4_, 1 g/L NaF, 4 g/L C_4_H_6_O_6_Na_2_, 6 g/L NaNO_3_, + 0.5 g/L of Basic bath TSPP or ATMP or EDTA	n/a	20 min, 45 °C	n/a	E_corr_ shift from −1.45 V to −1.40 V, −1.43 V and −1.45 V for basic phosphate solution to solution containing TSPP, ATMP and EDTA, respectively. I_corr_ reduction from 30 µA/cm^2^ to 8.5 µA/cm^2^, 10 µA/cm^2^and 28 µA/cm^2^ in the presence of TSPP, ATMP and EDTA, respectively.	Salt-water test (SWI), 3.5% NaCl	AZ91	[203] (2014)	
**Calcium-phosphate**	Grinding to 2000 grits, degreased in absolute acetone, rinsed by DI water, dried under atmospheric condition	40 g/L Ca(NO_3_)_2_, 40 mL/L H_3_PO_4_ then in the 5 g/L NaF solution, then surface modification in a 0.05 M ethanol of stearic acid solution	Phosphating 2.8; Fluoride 12	Phosphating: 20 min, 37 °C ± 2 °C fluoride bath: 2h, 80 °C, 15 h stearic acid	Micro-protrusions, submicro-lumps and nano-grains with diameter of about 1–2 µm. Ca_3_(PO_4_)_2_, Ca(H_2_PO_4_), Ca_10_(PO_4_)_6_F_2_, and MgF_2_	I_corr_ reduction from 129 µA/cm^2^ to 1.3 µA/cm^2^ for bare to the substrate coated with phosphate, fluoride and stearic acid. E_corr_ shift from −1.54 V to −1.36 V for bare to the substrate coated with phosphate, fluoride and stearic acid.	3.5% NaCl	Mg-5Zn-1.5Ca	[199] (2017)	
	Grinding to 2000 grit, cleaning in acetone. No pre-treatment such as alkaline degreasing or acid pickling.	12 g/L Ca(NO_3_)_2_.4H_2_O, 1.2 g/L CaO, 8 mL/L H_3_PO_4_ (85% v/v)	2.4–3.2	5 s–40 min, 15 °C, 37 °C, 60 °C	Ca_9_Mg(HPO_4_)(PO_4_)_6_, MgHPO_4_.3H_2_O. Thickest coating at bath pH of 3.2	Lowest i_corr_ 2.9 µA/cm^2^ obtained at pH 3.0	SBF solution	AZ60	[145] (2016)	
	Grinding to 2000 grits, cleaned in DI water and ethanol and then dried in open air	0.05 M Ca(NO_3_)_2_.4H_2_O, 0.03 M NaH_2_PO_4_.2H_2_O	n/a	48 h, room T	CaHPO_4_.2H_2_O, Ca_2_P_2_O_7_ (after heat treatment) with thickness of 30 µm	E_corr_ shifted from −1.666 V to −1.566 V and −1.515 V fore bare, Ca-P coated and Ca-P coated followed with heat treatment. I_corr_ reduced from 35 µA/cm^2^ to 3.5 µA/cm^2^ and 1 µA/cm^2^ for bare, Ca-P coated and Ca-P coated followed with heat treatment.	Hank solution	ZK60	[146] (2012)	
	Grinding to 300 grits, alkaline cleaning (NaOH, Na_3_PO_4_), acid pickling (CH_3_COOH+NaNO_3_) etching, HF activation, DI water	2 g/L Ce(NO_3_)_3_ 2 g/L La(NO_3_)_3_ 2 g/L KMnO_4_	4.0	5 min 40 °C	15 µm La_2_O_3_, CeO_2_, Mn_2_O_3_, and MnO_2_ homogeneous with microcracks	E_corr_ and i_corr_ decreased from 1.58 V/0.13 mA/cm^2^ to 1.44V/0.031 mA/cm^2^ for bare to RE coated substrate, compared to 1.11 V/0.056 mA/cm^2^ for Cr(VI) CC Excellent adhesion to substrate	3.5% NaCl	Mg–Li	[246] (2009)	
**Rare-earth element** **Rare-earth element**	Grinding to 600 grits, polishing by 1-µm diamond paste, acetone, distilled water, degreasing with NaOH and Na_3_PO_4_ at 80 °C, DI water	0.02 M Ce(NO_3_)_3_ 5 g/L H_2_O_2_ (30 wt.%)	4	15 min 25–55 °C	MgO, Mg(OH)_2_, CeO_2_, and Ce_2_O_3_, The highest uniformity and compactness of coating observed at 35 °C	E_corr_ and i_corr_ decreased from 1.543 V/0.25 mA/cm^2^ to 1.504 V/3 µA/cm^2^ for bare to RE coated substrate	3.5% NaCl	AZ91	[241] (2015)	Commercially available, high corrosion resistance / expensive, unsatisfactory long term stability
	Grinding to 2500 grits, polishing by 3.5-µm diamond paste, degreased in ethanol, acid pickling by HNO_3_ (0.8%) and then 40% HF, rinsed with distilled water and subsequent drying before each step	10 g/L Y(NO_3_)_3_	n/a	30 °C	Y_2_O_3_, YO_x/y_, Al_2_O_3_, and MgO	Improvement in corrosion resistance was not so significant, however, the post-treatment with the silica sol coating reduced the corrosion current density by two orders of magnitude, E_corr_ shifted positively about 140 mV, The corrosion current density decreased about two orders of magnitude	3.5% NaCl	AZ91	[219] (2017)	
	Grinding to 180 grits, cleaned with isopropyl, rinsed with DI water, dried in room temperature, etched in HNO_3_, alkaline cleaning in Na_2_SiO_3_.5H_2_O	4 wt.% CeCl_3_.7H_2_O, 6.7 wt.% H_2_O_2_, 0.25 wt.% organic gelatin	n/a	5–180 s in CeCC solution, followed by 5min at 85 °C immersion in 2.5wt.% NaH_2_PO_4_	Three-layer coating: nanocrystalline MgO, nanocrystalline CeCC and outer amorphous CeCC layer	Best corrosion behavior for the thinner CeCC (100 nm)	NSST	AZ31	[236] (2016)	
	Grinding to 1200 grits, rinsed with DI water, degreases with acetone, acid pickling in 0.15M HCl or 0.46 M HF, rinsed in DI water, dried in stream of air	0.05 M Ce(NO_3_)_3_.6H_2_O, 0.254 M H_2_O_2_	2.9	180 s, room T	200 nm thickness on HCl pickled samples with chemical composition of Mg(OH)_2_, Al(OH)_3_. 300 nm thickness HF-pickled samples contained MgF_2_, as well. CC contained Mg/Al hydroxide and CeO_2_	Adhesion grade was 1B, 3B, and 5B for the cerium coating on the as-polished AZ31, the HCl-pickled AZ31, and the HF-pickled AZ31, respectively. Corroded area after 24 h of SST was >80% for the cerium-coated as-polished AZ31, 20~25% for the cerium-coated HCL-pickled AZ31, and <1% for the cerium-coated HF-pickled AZ31.	SST, 3.5% NaCl for electrochemical tests, adhesion test according to ASTM D3359-02	AZ31	[408] (2012)	
	Grinding to 800 grits, Rinsed with DI water, dried in a stream of hot air,	0.05 M Al (NO_3_)_3_, 0.001–0.05 M Ce(NO_3_)_3_	n/a	2 min, 15–20 °C	6 µm compact coating with some observed micro-cracks, Al(OH)_3_, Al_2_O_3_, Mg(OH)_2_, MgO, Ce_2_O and Ce_2_O_3_	The most positive E_corr_ at Ce(NO_3_)_3_ concentration of 0.005 M, which also exhibited the lowest i_corr_ of value 0.022 mA/cm^2^	5 wt.% NaCl	AZ91	[409] (2013)	
	Grinding to 1500 grits, cleaning in acetone, degreased with NaOH+Na_3_PO_4_	6 g/L La(NO_3_)_3_, 3 g/L Na_2_MoO_4_	4	25 °C	5–6 µm, Cracked layer with “dry-mud” morphology	E_corr_ shifted 500 mV to more positive values with respect to bare substrate. two orders of magnitude in i_corr_	3.5 wt.% NaCl	AZ31	[229] (2010)	
	Grinding to 1000 grit, degreased in acetone, washed with triply distilled water	5–50 mM Ce(NO_3_^−^)_3_·6H_2_O in purified N_2_ gas saturated atmosphere. 1–20 mM H_2_O_2_, 1–10 mM ascorbic acid	n/a	50 °C	CeO, CeO_2_, Ce_2_O_3_, MgO, Mg(OH)_2_	Small positive shift of E_corr_ and 4-time reduction of i_corr_ by addition of ascorbic acid to the bath.	Ringer solution	AZ91	[221] (2016)	
**Vanadate**	Grinding to 2400 grits, ultrasonication in acetone, stream air drying	NaVO_3_ 30 g/L Vanadium oxide precipitation	8	10 min/ 80 °C	0.1–1.6 µm Vanadium oxides microcracks	E_corr_ and i_corr_ decreased from −1.63 V /0.1 mA/cm^2^ to −1.37 V/0.56 µA/cm^2^ for bare to vanadate coated alloy	0.1% NaCl	AZ61	[254] 2007	High corrosion protection ability for a number of mg alloys / toxic if swallowed, suspected of damaging fertility, toxic to aquatic life with long lasting effects [258]. not industrially feasible
	Grinding to 800 grits, acetone, air drying	NaVO_3_ 50 g/L Vanadium oxide precipitation		10 min/ RT	1.5–2.5 µm Vanadium oxides microcracks	n/a	3.5% NaCl	AZ31	[253] 2011	
**Molybdate**	Grinding to 300 grits, alkaline decreasing 40 g/L NaOH, 10 g/L Na_3_PO_4_·12H_2_O Acid pickling (200 mL/L CH_3_COOH 50g/L NaNO_3_)	25 g/L Na_2_MoO_4_·12H_2_O 4 g/L NaF (optional SiO_2_ nanoparticles)	3	10 min/ 66 °C	12 µm Multiple microcracks SiO_2_ addition decreases the number of microcracks	For MoO_4_^−^ −1.04 V/16.1 µA/cm^2^ For MoO_4_^−^+SiO_2_ −0.81 V/3.6 µA/cm^2^	3.5% NaCl	AZ31	[260] 2013	Moderate corrosion protection / limited commercial availability
**Stannate**	Grinding to 1500 grits, air drying, acid pickling and activation 0.25%HF + 0.25%HCl	0.25 M Na_2_SnO_3_, 0.073 M CH_3_COONa, 0.13 M Na_3_PO_4_, 0.05 M NaOH.	alkaline	1 h/ 40 °C potentiostatic conditions	0.6–1.8 µm deposit composed of MgSnO_3_·3H_2_O	E_corr_ decreased from −1.77 V to −1.55 V for bare to stannate treated alloy	Borate buffer (0.15 M H_3_BO_3_ and 0.05 M Na_2_B_4_O7, pH 8.5)	AZ91	[266] 2007	Commercially available, environmentally acceptable / moderate corrosion protection, long time treatment, typically requires elevated temperature
	Grinding to 2400 grits, polishing with 3 and 1um diamond paste, ultrasonication in acetone, stream hot air drying, activated in 11.25% HF	30–60 g/L K_2_SnO_3_·3H_2_O, 10 g/L CH_3_COONa·3H_2_O, 50 g/L Na_2_P_2_O_7_ 2.5–15 g/L NaOH Nucleation and growth of round particles	12.6–13.2	2–60 min/ 60–90 °C	Few microns thick round agglomerates of submicron particles with remaining discontinuity in surface coverage	SST with rating numbers varying from 8 (bare AZ61) to 4 (stannate treated AZ61)	5% NaCl SST	AZ61	[410] 2006	
	Grinding to 1000 grits, cold air stream drying, acid pickling and activation HF, HCl, HNO_3_	50 g/L K_2_SnO_3_, 10 g/L CH_3_COONa, 50 g/L Na_2_P_2_O_7_ 5 g/L KOH	12.4	1–10 min/ 82 °C	Round agglomerated submicron particles of MgSnO3·3H2O	E_corr_ and i_corr_ decreased from -1.60 V /12 µA/cm^2^ to –1.44 V/0.67 µA/cm^2^ for bare to stannate treated alloy	0.05 M NaCl + 0.1 M Na_2_SO_4_	AZ91	[86] 2011	
	Grinding to 800 grits, acetone, air drying	25 g/L K_2_SnO_3_·3H_2_O + NaOH	12.9	30 min/ RT		Corrosion rate decreased by 1/3–1/2	3.5% NaCl	AZ91	[411] 2013	
**Fluoride**	Untreated	7–28 M HF	Highly acidic	1–24 h/ RT	Up to 2 µm Mg(OH)_x_F_2__−x_	E_corr_ and i_corr_ decreased from −1.473 V/0.11 mA/cm^2^ to −1.468 V/0.017 mA/cm^2^ for bare to HF treated alloy	3.5% NaCl	AZ31	[99] 2010	Commercially available / moderate corrosion protection HF is highly toxic, fatal if swallowed, in contact with skin or if inhaled [293]
**Fluoro-metallates** **Zr, Ti or Zr/Ti fluorides**	Grinding to 1200 grits	0.01 M TiCl_4_, 0.01 M H_2_SiF_6_ 5 mL/L HNO_3_	4 by NaOH	0.5–10 min/ 40 °C	0.2–0.5 µm micro-cracks Mg(OH)_2_, MgF_2_, Si(OH)_4_, Ti(OH)_4_	E_corr_ and i_corr_ decreased from −1.55 V/9.9 µA/cm^2^ to −1.48 V/0.48 µA/cm^2^ for bare to treated alloy	0.05 M NaCl 0.1 M Na_2_SO_4_; SST	AZ31	[125] 2012	Commercially available, excellent paint adhesion, good corrosion resistance, Single step process, operable at room temperature, Well-compatible with pre- and post-treatment / requires accurate process operation, active R&D topic
	Grinding to 2000 grits, ultrasonication in acetone, hot air drying, 20% HF 20 h/RT	0.2 M Zr(NO_3_)_4_: methanol:AcAc (molar 1:4:8) aged for 48 h, concluded by alloy dipping		Withdrawal speed: 6 m/h	Micron and submicron pores and cracks	E_corr_ and i_corr_ decreased from −1.614 V/12.9 µA/cm^2^ to −1.516 V/0.53 µA/cm^2^ for bare to treated alloy	3.5% NaCl	AZ91	[289] 2008	
	Grinding to 2000 grits, degreasing in NaOH (40 g/L) +Na_2_SiO_3_ (40 g/L)	(a) H_2_TiF_6_ 0.5 g/L and H_2_ZrF_6_ 1.5 g/L (b) H_2_ZrF_6_ 1.5 g/L + tannic acid 1.5 g/L (c) H_2_TiF_6_ 0.5 g/L + tannic acid 1.5 g/L	2.5 by NaOH	3 min/ 25–30 °C	5–6 μm Micro-cracks MgF_2_, Mg(OH)_2_, MgO, TiO_2_, ZrO_2_, Ti, and Zr metal–organic complex	i_corr_ decreased from 93.72 μA/cm^2^ to 1.047 μA/cm^2^	3.5 wt.% NaCl	AZ91	[306] 2015	
	Grinding for SST: alkaline (NaOH, Na_2_CO_3_, Na_3_PO_4_, soap) and acidic (NH_4_F, H_3_PO_4_) treatment followed by air drying	0.03–0.1 M Ce(NO_3_)_3_ 0.03–0.1 M ZrO(NO_3_)_2_ 0.02–0.05 M Nb_x_O_y_F_z_	4 by NH_4_F	24 h /RT	CeO_2_, Ce_2_O_3_, ZrO_2_, Nb_2_O_5_, MgO, MgF_2_, composition did not change after anodic/cathodic polarization	E_corr_ and i_corr_ decreased from –2.07 V/626 µA/cm^2^ to –1.76 V/13 A/cm^2^ for bare to 24 h treated alloy	0.5 M Na_2_SO_4_, or SST	AZ91	[296] 2008	
**Phytic acid** **(phytate)**	Grinding to 1400 grits, ultrasonication in acetone for 10 min	Phytic acid/0.5 g/L	5	20 min/RT	4–5 µm, Mg/Al phytate microcrackes	E_corr_ shifted from –1.906 V to –1.735 V i_corr_ decreased from 429.4 to 373.0 mA/cm^2^ for bare to PA coated substrate excellent adhesion to substrate and epoxy coating	5% NaCl	AZ61	[318]	Excellent adhesion to substrate and epoxy coating, environmentally benign / limited corrosion protection, relatively expensive, commercially unavailable
	Grinding to 1200 grits, DI water	Phytic acid/0.5%/chemisorption	5	10–30 min/ 29 °C	14–20 µm magnesium phytate (Mg_12−x_ H_x_Phy)	i_corr_ 37 µA/cm^2^ excellent adhesion	Phosphate buffer solution. pH 7.4 37 °C	AZ31	[324]	
	Grinding to 1500 grits, alkaline degreasing and acid pickling	Phytic acid/ 20 g/L/ deposition	6	10 min/ 35 °C	7 µm Mg/Al phytate, macroscopic: smooth gray microscopic: flower-like cracked deposits	E_corr_ shifted from −1.645 V to −0.905 V i_corr_ decreased from 1.1 mA/cm^2^ to 2.3 µA/cm^2^ for bare to PA coated substrate excellent adhesion	3.5% NaCl	Mg-Li alloy Mg - 11 wt.% Li, 3 wt.% Al, 0.5 wt.% RE	[322]	
	Grinding to 2000 grits, alkaline degreasing and acid pickling	Phytic acid / 20 g/L/ deposition	9–10	0.5–3 min 25 ± 5 °C, Then hot air drying	transparent, microcracks	E_corr_ shifted from −1.46 V to –1.31 V	0.05 M NaCl	AZ31	[412]	
	Grinding to 2000 grits, washing in acetone and DI water, hot air drying	Phytic acid/ 5 g/L/ deposition	8	20 min RT	0.34 µm Integrated and uniform	i_corr_ 6 orders of magnitude lower than bare alloy	RT 3.5% NaCl	AZ91	[323]	
	Grinding to 1200 grits, washing in acetone	Phytic acid/ 50% heat post-treatment improves corrosion resistance	n/a	3 h RT	2.2 µm	E_corr_ shifted from −1.64 V to –1.50 V i_corr_ decreased from 24 µA/cm^2^ to 1.2 µA/cm^2^ for bare to PA coated substrate excellent adhesion	Phosphate buffer solution	Pure Mg	[413]	
	Grinding to 4000 grits, washing in acetone, ethanol and DI water, followed by alkaline degreasing and acid pickling, followed by acetone, alcohol and DI water and hot air drying. Then 3M NaOH for 12 h at 60 °C, washed in DI water and dried in vacuum oven	Phytic acid/ 5 g/L covalent immobilization	5	20 min 60 °C	n/a	E_corr_ shifted from −1.44 V to –1.45 V i_corr_ decreased from 0.27 mA/cm^2^ to 0.14 mA/cm^2^ for bare to PA coated substrate excellent adhesion	Phosphate buffer solution 37 °C	Pure Mg	[60]	
	Grinding to 2000 grits, ultrasonic treatment in ethanol, dried by warm air	Slurry prepared at 55 °C for 48 h 0.06 M Mg(NO_3_)_2_·6H_2_O, 0.03 M Al(NO_3_)_3_·9H_2_O +0.06 M Na_2_MoO_4_ and 0.2 M NaOH	alkaline	AZ31 sample was kept in slurry for 36 h 100 °C in autoclave	17 µm Typical LDH flakes, MgAl-LDH, (Mg_6_Al_2_(OH) _16_MoO_4_·4H_2_O)	E_corr_ shifted from −1.54 V to –1.21 V i_corr_ decreased from 31.7 µA/cm^2^ to 0.16 µA/cm^2^ for bare to LDH coated alloy	3.5% NaCl	AZ31	[384] 2014	
**LDH**	Grinding to 2000 grits, ultrasonic treatment in ethanol, dried in air stream	Slurry prepared at 40 °C for 48 h + 12 h [Mg(NO_3_)_2_ Al(NO_3_)_3_ at molar ratio 3:1 + Na_2_CO_3_/NaOH	alkaline	AZ31 sample was kept in slurry for 24–48 h 100 °C in autoclave	7 µm typical LDH flakes, MgAl-LDH, (Mg6Al_2_(OH)_16_CO_3_·4H_2_O)	E_corr_ shifted from –1.56 V to –1.18 V i_corr_ decreased from 30.4 µA/cm^2^ to 0.07 µA/cm^2^ for bare to LDH coated alloy	3.5% NaCl	AZ31	[377] 2014	High corrosion protective ability, environmentally benign, can be loaded with corrosion inhibitors for active corrosion protection, can be grown at RT and ambient pressure / at the early development stage, relatively expensive, active R&D topic
	Grinding to 5000 grits, PEO treatment, Ultrasonic treatment in ethanol, dried in air stream	0.1 M NaNO_3_	8 by NaOH	12 h 100 °C in autoclave	8 µm typical LDH flakes, LDH-MgAl-NO_3_ or LDH-MgAl-VO_3_	E_corr_ shifted from –0.74 V to –0.47 V i_corr_ decreased from 3.9 µA/cm^2^ to 0.95 µA/cm^2^ for PEO treated to PEO-LDH-NO_3_ coated alloy	3.5% NaCl	AZ31	[369] 2017	
	Grinding to 5000 grits, PEO treatment, Ultrasonic treatment in ethanol, dried in air stream	0.05 M Al(NO_3_)_3_, 0.3 M NH_4_NO_3_	8.72–12.04	12 h 100 °C in autoclave	Typical LDH flakes, MgAl-LDH, Mg(OH)_2_	E_corr_ shifted from −1.51 V to −1.34 V, i_corr_ shifted from 32.68 to 0.118 μA/cm^2^ for bath pH 8.72 to 11.72	3.5% NaCl	AZ31	[370] 2017	
	Grinding to 1200 grits, DI water, dried in air	Al(NO_3_)_3_ EDTA, NTA	8–12	15 min to 6 h at 95 °C and 48 h at 25 °C ambient pressure	20–60 nm typical LDH flakes, MgAl-LDH	n/a	n/a	AZ91	[385] 2018	
	PEO-treated AZ91 PEO electrolyte 1 g/L KOH, 8 g/L Na_3_PO_4_ and 12 g/L NaAlO_2_	0.05 M Al(NO_3_)_3_, 0.5 M NaNO_3_, 0.5 g of AZ91 flakes 0.05 M DTPA 0.003 M salicylate-Na	10.0	0.5 to 8 h at 70 or 95 °C, ambient pressure	Typical LDH flakes were grown on top of PEO and inside PEO pores	n/a	n/a	AZ91	[373] 2020	

## 4. Summary and Perspective

Scientific research is being actively carried out in industry and academia to find alternative conversion coatings for the hazardous chromate conversion coatings. Various types of conversion coatings have been intensively investigated to mimic the unique self-healing characteristic of CCC and achieve a comparable corrosion protection performance. In spite of numerous reports of conversion coatings with self-healing properties, a systematic comparison of the performance of conversion coatings with CCCs has barely been conducted.

Given that Cr_2_O_3_ is one of the main components of the chromate conversion coating, Cr(III) could be considered as a replacement for CCC. However, less corrosion resistance has been observed for Cr(III) [123] due to the deposition of a thinner layer. Some reports also claim that Cr(III) conversion layers contain the banned Cr(VI) [414]. Cr(III) conversion coatings have rarely been studied on magnesium alloys and they undeniably merit further attention. 

The phosphatization of metals is a well-known process that has also been intensively studied on Mg alloys. Different approaches such as the addition of zinc to the phosphatizing bath or hydrothermal post-treatments may yield a PCC with superior corrosion resistance. Recent studies have put efforts into designing PCCs with self-healing properties. However, the majority of the reports on PCC are currently dedicated to bioapplications and an evaluation of their performance in comparison to CCCs is missing. 

Although phosphate-based conversion coatings are widely used in industry, there is a tendency for industry to switch to phosphate-free surface treatment technologies such as thin film hexafluoro-Zr/Ti based conversion coatings. Arguments for customers are usually savings: energy savings, cost savings, raw material savings, workforce savings, hardware savings, better health protection for staff, etc. Although the stand-alone conversion coating based on hexafluoro-Ti/Zr may not provide superior barrier properties, the strong adhesion promotion feature of these coatings is a decisive factor for industrial applications. Thus, further development and fundamental studies on these conversion coatings, specifically for Mg alloys, are highly appreciated.

The rare earth conversion coatings can be regarded as a solution that provides an effective barrier layer against the corrosive media. However, factors such as high cost, lack of self-healing ability, and common low adhesion to the subsequent polymer coating have made it difficult for them to provide a cost-effective alternative to CCCs. 

The self-healing effect of vanadium-based conversion coatings similar to CCCs yields a corrosion protection performance that is comparable to CCCs [123]. However, the high efficiency of the vanadium coating is offset by the high toxicity of vanadate compounds comparable to that of chromates.

Organic-based conversion coatings containing chelating compounds with multi-ligands such as phytic acid and tannic acid are also another formulation to offer new mechanisms of corrosion protection. When combined with other inorganic-based conversion coatings, the few reports on the accomplished self-healing of Mg substrates make it worthwhile to investigate. Moreover, the free functional groups of such organic molecules are capable of providing strong adhesion to subsequent polymer coatings. 

Other organic-based conversion coatings such as ionic liquid-based conversion coatings and deep eutectic solvents also reportedly provide some level of protection against corrosion. However, limited by their high cost and difficult handling, only a few reports with unsatisfactory performance are available. Nevertheless, the practically unlimited number of potential organic molecules and their combinations represent an undeniable territory for investigation. In this case, the use of artificial intelligence-driven predictive methods signifies an inescapable avenue to explore effective formulations. 

LDH conversion coatings, due to their ability to release customized inhibitors on demand and trap corrosive species, are highly promising coatings on the route to endowing protection methods with self-healing abilities. A new method that allows for the formation of LDH at room temperature and ambient pressure has recently been reported [373,385]. It involves the addition of Mg^2+^ and Al^3+^ chelating agents (the simplest example being EDTA) that controls the amount of free Mg^2+^ and Al^3+^ in the solution and fosters the formation of LDH in carbonate free electrolytes. Notably, this represents a significant step toward overcoming the technological limitations of direct LDH growth on magnesium alloys. Once mature, LDH, as an intrinsic conversion product, might emerge as a strong candidate for chromate replacements. 

The achievement of an effective conversion coating against corrosion has a strong linkage with a proper pre-treatment on the Mg substrate. The Mg surface condition is of crucial importance to achieve the desired properties from some conversion coatings such as hexafluoro-Zr/Ti-based thin films. The majority of newly developed conversion coatings follow the already established pre-treatment methods. However, the remarkable effects of recent pre-treatment methods, which include the modification of surface electrochemical properties using complexing agents to enhance the subsequent phosphate conversion coating performance [110], have demonstrated the overlooked influence of pre-treatment methods worthy of investigation. Approaches that are more systematic are necessary toward understanding the general mechanisms of coating formation and the detailed influence of bath parameters. The involvement of high-throughput testing and machine learning for data analysis and the identification of hidden trends will be highly beneficial.

## Data Availability

Not applicable.
